# Evaluation of pulmonary single‐cell identity specificity in scRNA‐seq analysis

**DOI:** 10.1002/ctm2.1132

**Published:** 2022-12-10

**Authors:** Xuanqi Liu, Guang Xu, Chengshui Chen, Yuanlin Song, Wei Wang, Xiangdong Wang

**Affiliations:** ^1^ Department of Pulmonary and Critical Care Medicine Zhongshan Hospital Fudan University Shanghai Medical College Shanghai China; ^2^ Shanghai Institute of Clinical Bioinformatics Shanghai China; ^3^ Shanghai Engineering Research Center for AI Technology for Cardiopulmonary Diseases Shanghai China; ^4^ College of Computer Science Fudan University Shanghai China; ^5^ Department of Respiratory Medicine The First Hospital of Wenzhou Medical University Wenzhou China; ^6^ Quzhou Hospital of Wenzhou Medical University Quzhou China

Dear Editor,

The single cell RNA sequencing (scRNA‐seq) technology provides new insights into understanding of single‐cell transcriptomic atlas and intercellular communication.^1^ scRNA‐seq is used to characterize the considerable heterogeneity and complexity of cell type, to uncover the cell fate and context, critical molecular features and progression trajectories, as well as to explore potential pathogenesis and individualized therapeutic targets.^2^, ^3^, ^4^ One of major challenges is to identify of biology‐specific biomarkers as the cell identity for cell phenotypes and functional sub‐types, although bioinformatic and fstatistical methods for scRNA‐seq are developed and improved rapidly.^5^ More and more cell type/subtypes are identified in response to various stimulus, external challenge and even pathological conditions. The correctness and specificity of cell‐specific transcriptomic profiles based on scRNA‐seq are highly dependent upon the accuracy of cell type identity and cellular annotation. The process of mapping single cell atlas is based on selected marker genes. Intricated cell types and subtypes are defined by the provided cell annotations and further validated using cell identity marker gene panels (ciMGPs),^6^ so the specificity of ciMGPs is critical to construct the single‐cell profile.

The current studies aim at evaluating the specificity of ciMGPs for various pulmonary single‐cell identities and to define disease‐specific alterations of single cell populations labelled with ciMGPs. We screened and selected 57 ciMGPs from previous studies^6^, ^7^, ^8^ and validated the identities of ciMGPs‐based cell types/subtypes in lung tissues from healthy subjects or patients with idiopathic pulmonary fibrosis (IPF), chronic obstructive pulmonary disease (COPD), systemic sclerosis (SSC), lung adenocarcinoma (LUAD), large cell cancer (LCC) or para‐cancer tissues as pair‐controls, as detailed in Supplemental Materials. Of those cells, immune cells resident in the lung tissue included nine subtypes of lymphoid cells and 15 subtypes of myeloid cells,^8^ and lung parenchymal cells had 15 subtypes of epithelia, nine of endothelia and nine of stromal cells, as presented in Figures S1–S48 and details in Tables S6–S11. We comprehensively assessed and quantified the specificity of each ciMGP representing 57‐specific cell subtypes among lung diseases. We firstly developed the criteria and schematic diagram to determine the specificity and accuracy of lung cell ciMGPs, as explained in Supplemental Method. The scRNA‐seq data for evaluation were collected from various databases (Tables S1–S3). We defined the overlap expression rate (OER) of ciMGPs in a cell subtype was less than 5%, as the cell‐specific marker panel with high specificity, when compared with the expression in other cell types/subtypes, between 5% and 10% as the ‘cell‐associated marker panel’ with moderate specificity, or more than 10% as ‘cell‐reference marker panel’ with low specificity, as explained in Supplemental Method and Figure 5. We dedicate special attention to the alteration of ciMGP's specificity in illness states, which presents profound insight into the clinical promotion and popularization.

We established the transcriptomic profiles of pulmonary single cells based on unified manifold approximation and projection (UMAP), reflecting the abundance and distribution of cell types and subtypes (Figure 1). The specificity of ciMGPs was evaluated in different cell types, subtypes, locations and diseases. The OER values of B cell (Figure 2A) and adventitial fibroblast (Figure 2B) were less than 5% in normal lung tissue or various lung diseases.

**FIGURE 1:**
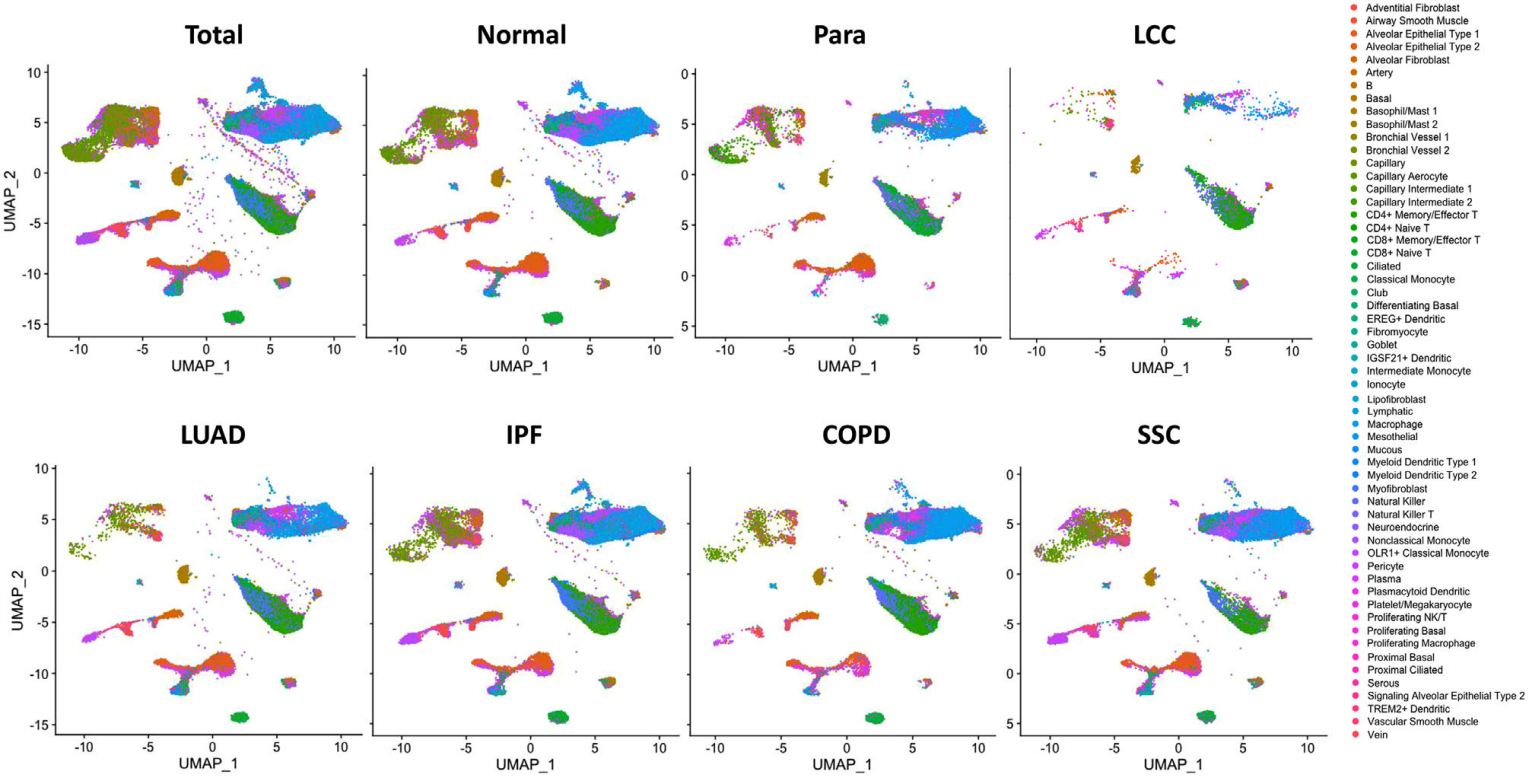
The uniform manifold approximation and projection (UMAP) representation of the lung cell landscape. Unsupervised analysis of 57 pulmonary single‐cells including 15 subtypes of epithelial cell, nine of endothelial cells, nine of stromal cells and 24 of immune cells harvested from lung tissues of healthy controls (normal) or patients with chronic obstructive pulmonary disease (COPD), idiopathic pulmonary fibrosis (IPF), lung adenocarcinoma (LUAD), large cell lung cancer (LCC), para‐tumor tissue (Para) and systemic sclerosis (SSC) and their corresponding characterization.

**FIGURE 2:**
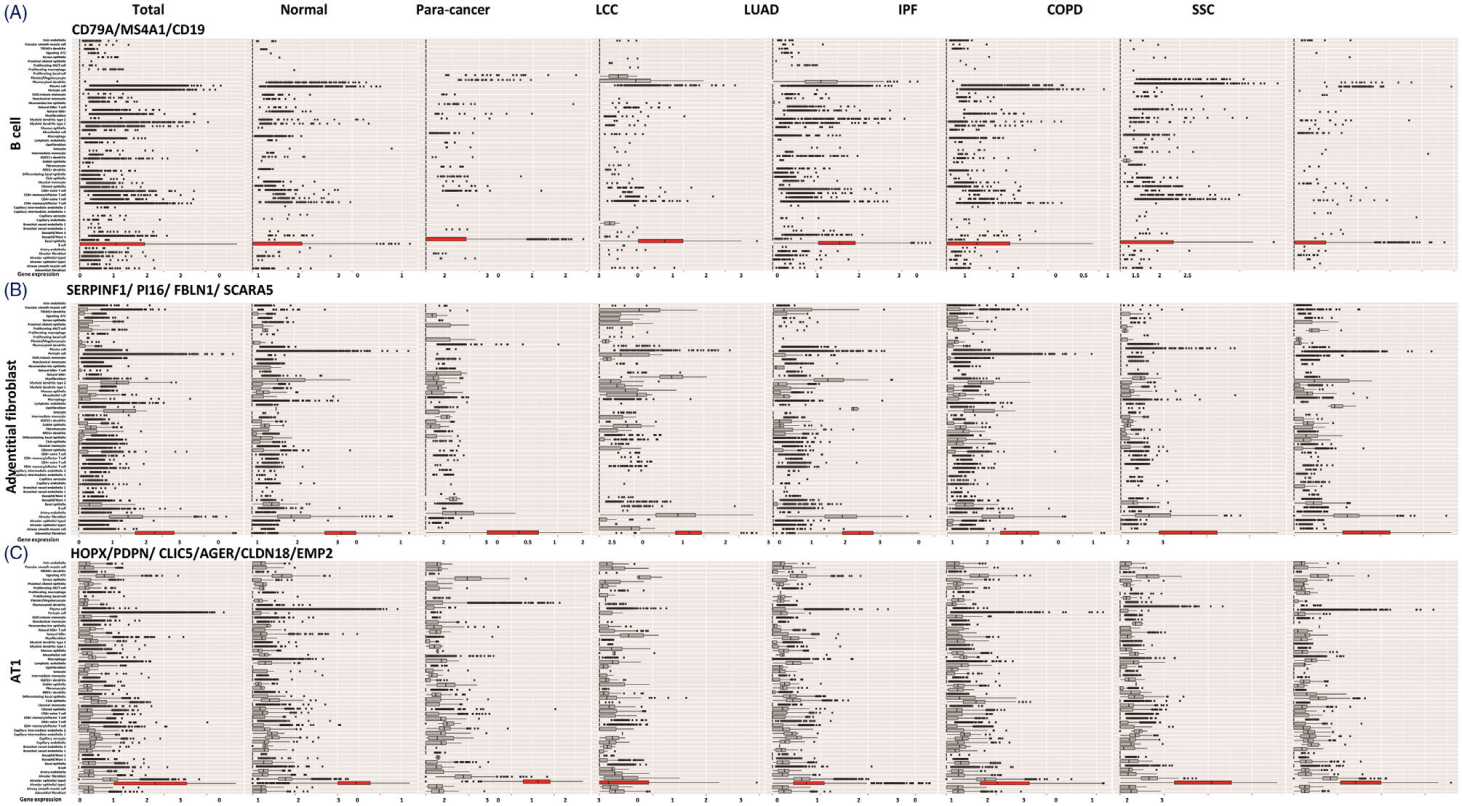
Cell‐specific panel in lung diseases. The box plot indicated the average mRNA expression of the panel in B cell (A; CD79A, MS4A1, CD19), the panel in adventitial fibroblast (B; SERPINF1, PI16, FBLN1, SCRAR5) and the panel in AT1 (C; HOPX, PDPN, CLIC5, AGER, CLDN18, EMP2) in 57 cell types from normal lung samples and six lung diseases (samples of para‐cancer, LCC, LUAD, IPF, COPD, SSC). The RED bars represent panels of B cell, AT1 and adventitial fibroblast, as the cell‐specific panel with <5% overlap expression rate as compared to other cell subtypes. The detailed calculation procedure of overlap expression rate can be seen in the Supplementary Methods.

The AT1 ciMGPs showed highly specific in most samples except for LCC, as compared with the remaining 56 cell subtypes (Figure 2C). Of 15 subtypes of lung epithelial cells, the ciMGPs specificity of ciliated cell was the highest in eight lung tissues (Table 1). The basal epithelia failed to be detected in LUAD samples based on the provided panel, and the efficacy of panel in goblet epithelia was weakened in LCC samples, according to the criteria proposed (Table 1). The specificity of ciMGPs representing pericyte cells and artery endothelial cells were comparatively decreased in the states of LCC and SSC (Table 1, Figures S4 and S35). However, ciMGPs mRNA expression of bronchial vessel 2 and pulmonary inocyte was hardly detected in all kinds of cells and lung diseases in our research (Table 1, Figures S8 and S24). The representatives of cell‐specific panels in the process of cell annotation were summarized and presented in Tables S4 and S5.

**TABLE 1:** ‘Overlap expression cell subset rate’ of cell subset‐specific, cell subset‐associated, cell subset‐reference mark gene panels of human lung tissues harvested from patients with lung adenocarcinoma (LUAD), large cell cancer (LCC), idiopathic pulmonary fibrosis (IPF), chronic obstructive pulmonary disease (COPD), and systemic sclerosis (SSC) total, normal (norm), and para‐cancer human lung tissues

Cell type	Total	Normal	Para‐cancer	LCC	LUAD	IPF	COPD	SSC	Cell‐specific (<5%)	Cell‐associated (5%–10%)	Cell‐reference (>10%)
Adventitial fibroblast	0	0	0	3.57	3.57	1.79	0	1.79	8	0	0
Airway smooth muscle cell	1.79	3.57	0	1.79	0	1.79	1.79	1.79	8	0	0
Alveolar epithelial type 1	0	0	0	41.07	5.36	1.79	0	0	6	1	1
Alveolar epithelial type 2	1.79	1.79	1.79	5.36	1.79	1.79	1.79	1.79	7	1	0
Alveolar fibroblast	1.79	3.57	0	17.86	1.79	1.79	0	1.79	8	0	0
Artery endothelia	0	0	0	3.57	0	0	0	1.79	8	0	0
B cell	0	0	0	1.79	1.79	0	0	0	8	0	0
Basal epithelia	1.79	3.57	0	0	ND	3.57	3.57	3.57	7	0	0
Basophil/mast 1	1.79	3.57	0	ND	ND	3.57	3.57	3.57	6	0	0
Basophil/mast 2	1.79	1.79	1.79	1.79	1.79	1.79	1.79	1.79	8	0	0
Bronchial vessel endothelia 1	33.93	26.79	ND	3.57	25	5.36	30.36	16.07	1	1	5
Bronchial vessel endothelia 2	ND	ND	ND	ND	ND	ND	ND	ND			
Capillary endothelia	8.93	7.14	5.36	14.29	14.29	23.21	7.14	3.57	1	4	3
Capillary aerocyte	0	1.79	1.79	0	0	0	1.79	1.79	8	0	0
Capillary Intermediate endothelia 1	8.93	5.36	3.57	ND	5.36	7.14	5.36	12.5	1	5	1
Capillary intermediate endothelia 2	30.36	23.21	28.57	21.43	26.79	37.5	23.21	30.36	0	0	8
CD4+ memory/effector T cell	28.57	35.71	21.43	21.43	12.5	41.07	85.71	25	0	0	8
CD4+ naive T cell	73.21	76.79	60.71	14.29	14.29	91.07	76.79	62.5	0	0	8
CD8+ memory/effector T cell	10.71	10.71	10.71	10.71	10.71	10.71	10.71	8.93	0	1	7
CD8+ Naive T cell	8.93	5.36	8.93	8.93	8.93	7.14	7.14	10.71	0	7	1
Ciliated epithelia	3.57	1.79	0	0	0	3.57	3.57	3.57	8	0	0
Classical monocyte	5.36	3.57	1.79	3.57	3.57	5.36	1.79	1.79	6	2	0
Club epithelia	10.71	5.36	1.79	67.86	12.5	7.14	5.36	7.14	1	4	3
Differentiating basal epithelia	14.29	19.64	ND	17.86	14.29	17.86	23.21	21.43	0	0	7
EREG+ dendritic	12.5	14.29	10.71	10.71	7.14	14.29	10.71	10.71	0	1	7
Fibromyocyte	5.36	7.14	ND	5.36	5.36	5.36	12.5	7.14	0	6	1
Goblet epithelia	3.57	0	1.79	5.36	3.57	3.57	1.79	3.57	7	1	0
IGSF21+ dendritic	16.07	16.07	10.71	10.71	14.29	16.07	14.29	16.07	0	0	8
Intermediate monocyte	25	26.79	28.57	14.29	16.07	21.43	25	19.64	0	0	8
Ionocyte	ND	ND	ND	ND	ND	ND	ND	ND			
Lipofibroblast	78.57	ND	ND	ND	89.29	96.43	ND	42.86	0	0	4
Lymphatic endothelia	0	0	0	12.5	0	0	0	0	7	1	0
Macrophage	5.36	7.14	0	14.29	5.36	7.14	3.57	8.93	2	5	1
Mesothelial cell	1.79	0	1.79	21.43	12.5	0	0	ND	5	0	2
Mucous epithelia	3.57	3.57	3.57	5.36	3.57	3.57	1.79	3.57	7	1	0
Myeloid dendritic type 1	8.93	8.93	10.71	8.93	7.14	8.93	10.71	8.93	6	2	0
Myeloid dendritic type 2	35.71	23.21	21.43	33.93	30.36	37.5	30.36	16.07	0	0	8
Myofibroblast	7.14	8.93	3.57	7.14	8.93	10.71	12.5	7.14	1	5	2
Natural killer cell	35.71	35.71	32.14	57.14	33.93	35.71	64.29	26.79	0	0	8
Natural killer T cell	44.64	37.5	35.71	32.14	35.71	44.64	39.29	41.07	0	0	8
Neuroendocrine epithelia	0	0	ND	ND	ND	ND	0	ND	3	0	0
Non‐classical monocyte	94.64	85.71	67.86	23.21	94.64	96.43	42.86	91.07	0	0	1
OLR1+ classical monocyte	21.43	21.43	14.29	5.36	16.07	23.21	17.86	21.43	0	1	7
Pericyte cell	0	0	0	1.79	0	0	0	1.79	8	0	0
0Plasma cell	ND	ND	ND	ND	ND	ND	ND	ND			
Plasmacytoid dendritic	ND	ND	12.5	ND	ND	ND	ND	ND	0	0	1
Platelet/Megakaryocyte	26.79	28.57	ND	ND	19.64	25	ND	26.79	0	0	5
Proliferating basal epithelia	ND	ND	ND	ND	ND	ND	ND	ND			
Proliferating macrophage	10.71	8.93	ND	ND	ND	8.93	ND	16.07	0	2	2
Proliferating NK/T cell	46.43	58.93	50	37.5	41.07	44.67	53.57	ND	0	0	7
Proximal basal epithelia	1.79	5.36	ND	ND	ND	3.57	3.57	3.57	4	1	0
Proximal ciliated epithelia	1.79	1.79	ND	ND	ND	1.79	3.57	3.57	5	0	0
Serous epithelia	ND	ND	0	3.57	7.14	ND	7.14	ND	2	2	0
Signaling alveolar epithelial type 2	1.79	1.79	1.79	0	48.21	7.14	1.79	1.79	6	1	1
TREM2+ dendritic	14.29	23.21	25	14.29	16.07	17.86	25	17.86	0	0	8
Vascular smooth muscle cell	3.57	5.36	3.57	1.79	3.57	3.57	1.79	3.57	7	1	0
Vein endothelia	21.43	8.93	1.79	35.71	16.07	17.86	17.86	14.29	1	1	06
Cell‐specific (<5%)	22	21	26	14	15	20	24	22			
Cell‐associated (5%–10%)	8	12	2	8	9	10	5	6			
Cell‐reference (>10%)	21	17	16	22	22	20	20	20			

The specificity and importance of ciMGPs can be dynamically changed on basis of disease nature and severity. The specificity of ciMGPs appeared in myeloid cells (Figure 3C) was higher than lymphoid cells (Figures 2A and 4B). The quality of ciMGPs of signaling AT2, CD8 naïve T cell and classic monocyte were considered as ‘cell‐associated panels’ in the normal lung tissue, while became the cluster of ‘cell‐specific panels’ in lung diseases and varied among lung diseases (Figure 3). The signaling AT2 panel clearly up‐expressed in AT2 from normal and para‐cancer lung tissues while became unclear in LUAD and IPF (Figure 3A). Compared with tissue‐resident cells, the ciMGP of immune cells, including CD4 memory effector T, CD4 naïve T, CD8 memory effector T, NK or NKT, showed relatively lower specificity, was more difficult to be annotated, meanwhile, and highly expressed in 2–3 other cell subtypes (Figures S12, S13 and S32, Table 1). It might be attributed to the relatively conserved function of structural cells in lungs, while immune cells exist in intermediate and functional states with continuously dynamical remodeling. Immune cells can be activated by external stimuli, to perform the primary force of host defense in lung, including the process of rapid recruitment and migration.^9^ Our data demonstrated that the ciMGPs specificity of capillary intermediate endothelia 2, natural killer and lipofibroblast was low in the majority of lung samples (Figure 4A–C), as the cell‐reference ciMGPs with high OERs. The specificity of ciMGPs has the value for deeply understanding the heterogeneity among various lung diseases and pathological states. Several ciMGPs with tissue‐specific pattern of expression have the potential of clinical implications.

**FIGURE 3:**
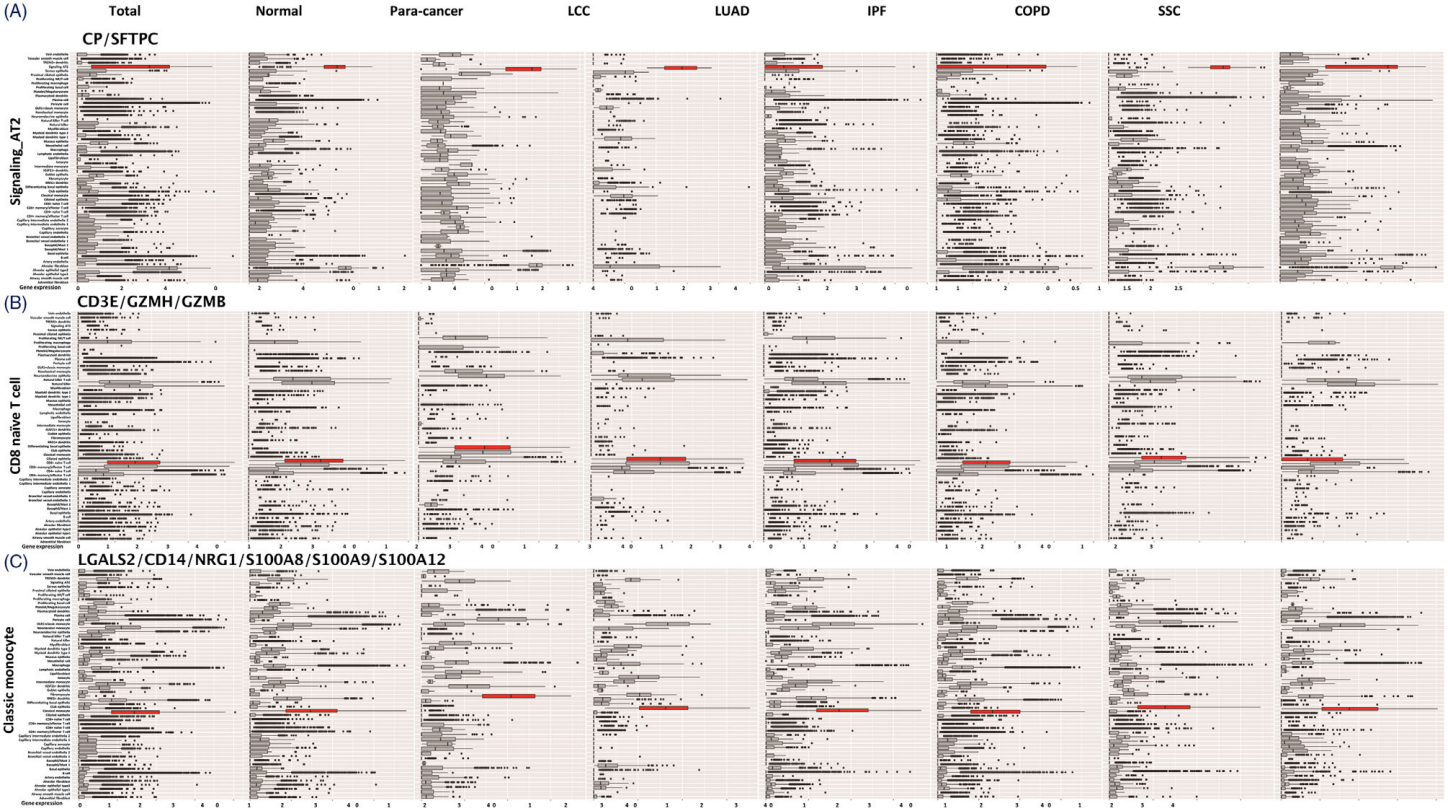
Cell‐associated panel in lung diseases. The box red plot represents the panel gene expression of signaling_AT2 (A; CP, SFTPC), CD8 naïve T cell (B; CD3E, GZMH, GZMB) and classic monocyte (C; LGALS2, CD14, NRG1, S100A8, S100A9, S100A12) harvested from normal lung samples and six lung diseases (samples of para‐cancer, LCC, LUAD, IPF, COPD, SSC). The panels of signaling_AT2, CD8 naïve T cell and classic monocyte were presented as examples of cell‐associated panel with 5%–10% overlap expression rate. The detailed calculation procedure of overlap expression rate can be seen in the supplemental methods.

**FIGURE 4:**
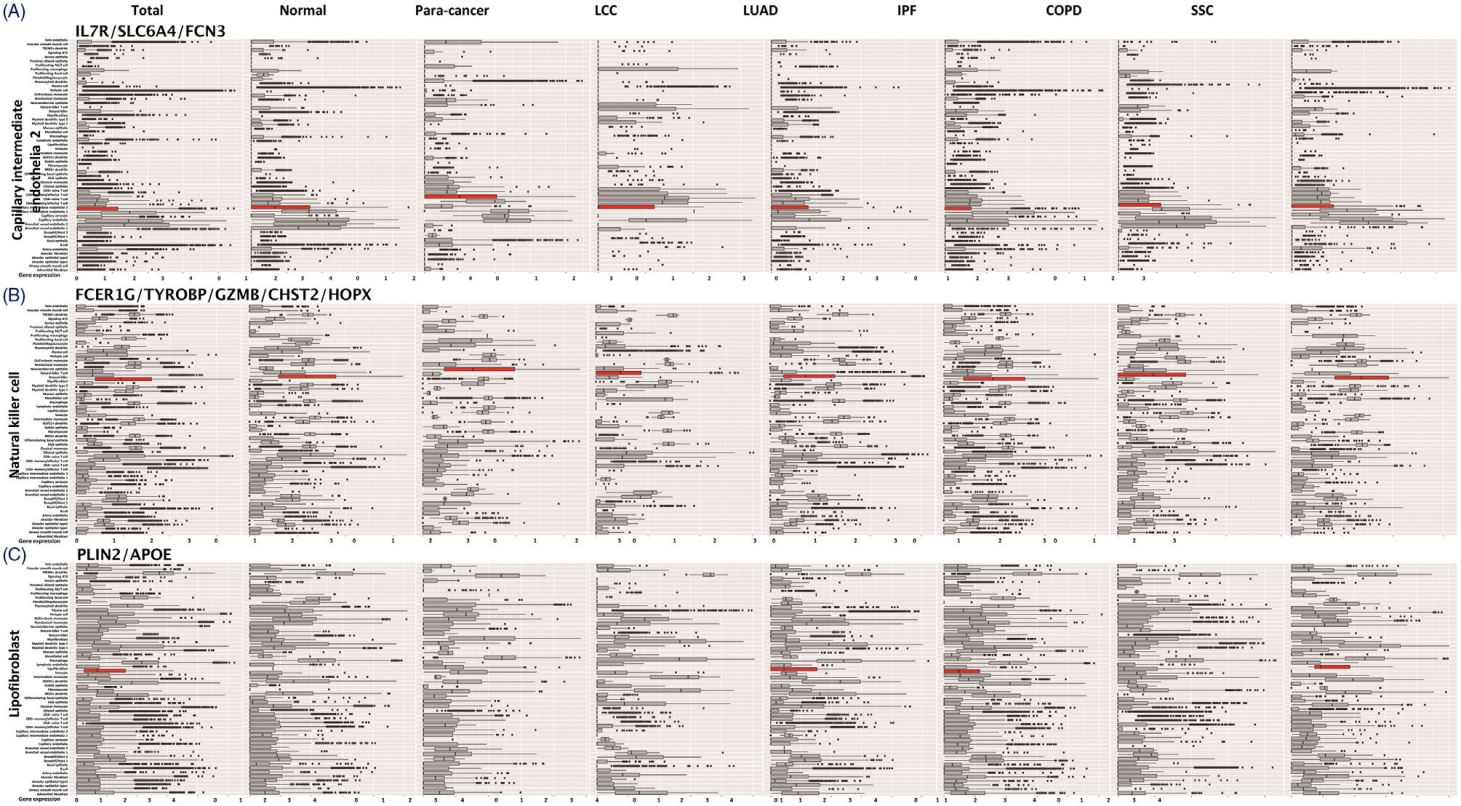
Cell‐reference panel in lung diseases. The box red plot indicated the panel gene expression in capillary intermediate endothelia 2 (A; IL7R, SLC6A4, FCN3), natural killer cell (B; FCER1G, TROBP, GZMB, CHST2, HOPX) and lipofibroblast (C; PLIN2, APOE) from normal lung samples and six lung diseases (samples of para‐cancer, LCC, LUAD, IPF, COPD, SSC). The panels of capillary intermediate endothelia 2, natural killer cell and lipofibroblast were presented as examples of cell‐reference panel with more than 10% overlap expression rate. The detailed calculation procedure of overlap expression rate can be seen in the Supplemental Methods.

**FIGURE 5:**
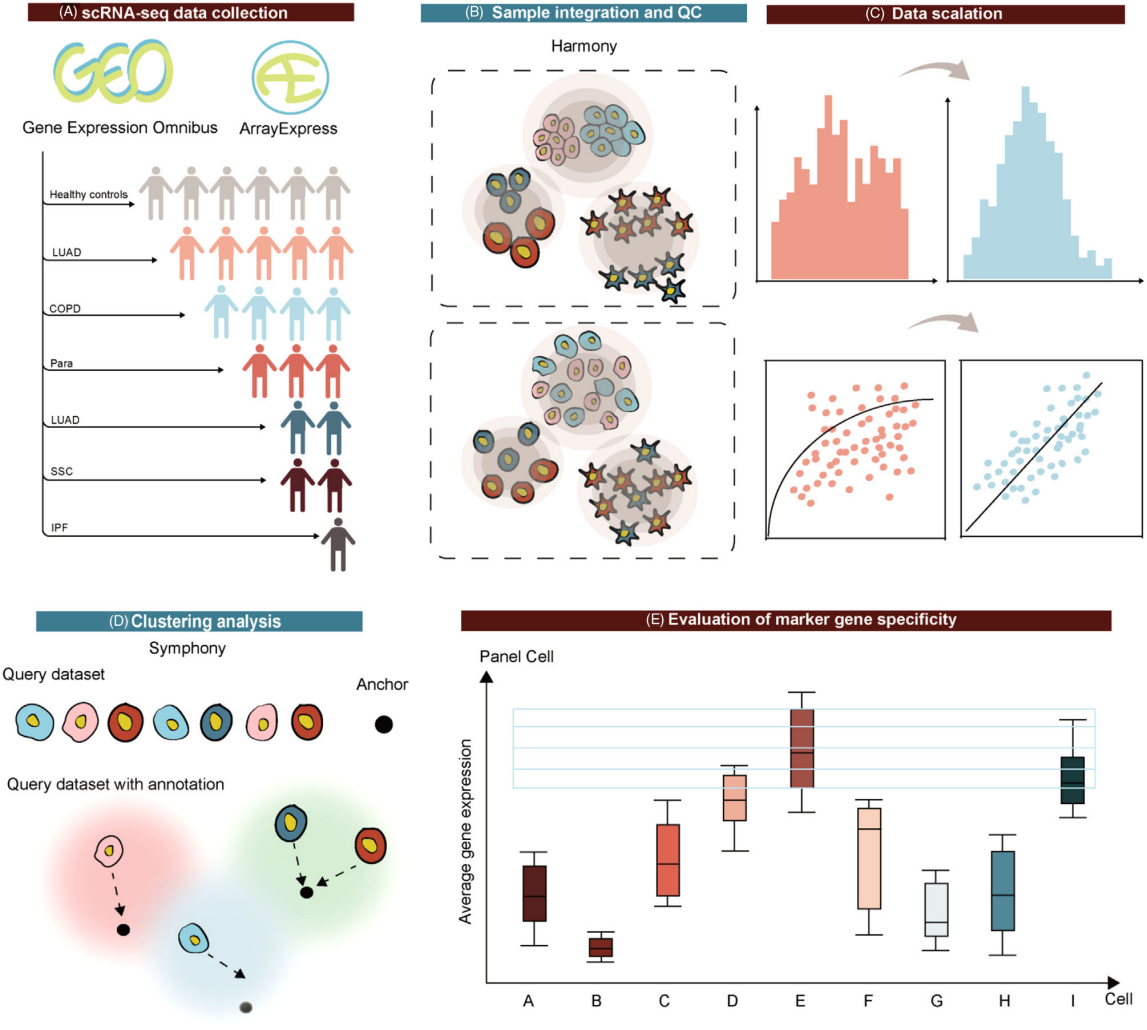
The flowchart showing the process of scRNA‐seq data collection (A), sample integration and quality control (B), data scalation (C), clustering analysis (D) and evaluation of marker gene panel specificity (E) among cells and diseases, based on the value of overlap expression rate (OER). OER is calculated from the overlapped marker cell subtype number/total cell subtype number *100%, and the indicator of the proportion ratio of MGP specificity in certain cell types/subtypes.

The ciMGPs of tissue‐resident cells and immune cells showed obvious differences in disease specificity, especially the subtypes of serous and CD4 naïve T cells, of which the specificity of ciMGPs was significantly higher in LUAD and LCC tissues (Table 1). The ciMGPs of AT1, Club or mesothelial cells showed comparatively low specificity in LCC samples (Table 1, Figures S17 and S27), while the ciMGPs of CD4 naïve T and non‐classic monocytes were higher in LCC (Table 1). Some ciMGPs expressed in multiple cell types, subtypes and diseases, for example, capillary intermediate endothelia 2 (Figure 4A), natural killer cells (Figure 4B) and lipofibroblast (Figure 4C).

It is critical to evaluate the specificity of ciMGPs in normal tissues to precisely define the representatives of cells and set the referenced baseline, in pathological disease tissues to check the abnormal values, and in development‐related cell subtypes to clarify the OERs. The cells with similar developmental‐lineage often share the common canonical molecular markers and resemble in their expression patterns, which makes it difficult to differ between AT1 and AT2, basophil/mast1 and 2, vascular and airway smooth muscle cells, proximal basal and basal cells, or myeloid dendritic type I and II. We found the variation range of ciMGPs among multiple cell subtypes and even types, for example, ciMGPs signature of dendritic cells also highly expressed in alveolar epithelial cells and basophil/mast cells (Figures S2, S5 and S6). It implied that they might share the common underlying gene expression pattern and close molecular interaction or crosstalk among diverse types of cells. The novelty of the present study is to comprehensively define and evaluate ciMGP specificity of pulmonary single‐cells and proposed the three categories using the OER values, to determine the difference of ciMGP specificity among multiple pathological conditions and to provide new alternatives for the quality control in scRNA‐sq data analysis and clinical application, as proposed previously.^10^ However, limited scRNAseq data set might impact the extrapolation of the conclusion. The specificity of distinct stages, surgery procedures, lesioned sites of lung diseases and even data derived from different sequencing methods needs to be further validated.

In conclusion, for the first time, we developed the criteria to evaluate the ciMGPs specificity of lung cell types/subtypes from various lung diseases and characterized three categories of cell‐specific, cell‐associated, and cell‐reference ciMGPs on basis of scRNA‐seq. The ciMGPs specificity varied among cell types and subtypes, disease natures and stages, as well as responses to therapies as the part of quality control in scRNA‐seq analysis, although the evaluation and criteria of ciMGPs need to be further improved and optimized. Thus, we believe that the precise evaluation of ciMGPs specificity is considerably important in bioinformatic analysis, single cell categories, data interpretations and accurate conclusion.

## CONFLICT OF INTEREST

The authors declare that there is no conflict of interest that could be perceived as prejudicing the impartiality of the research reported.

## Supporting information

Supporting Information

Supporting Information

Supporting Information

Supporting Information

Supporting Information

Supporting Information

Supporting Information

Supporting Information

Supporting Information

Supporting Information

Supporting Information

Supporting Information

Supporting Information
