# Supplementary material for: Evaluation of pulmonary single‐cell identity specificity in scRNA‐seq analysis
Source: Clin Transl Med. 2022 Dec 10;12(12):e1132. doi: 10.1002/ctm2.1132 (PMC9736794; doi:10.1002/ctm2.1132)

**Supplementary methods:**

**Pulmonary sc-RNA seq data collection** In the research, the pulmonary single cell RNA-sequencing (scRNA-seq) dataset is extracted and combined from 6 published scRNA-seq datasets: GSE128169 (Valenzi, E., 2019), GSE128033 (Morse, C., 2019), GSE136831 (Adams, T. S., 2020), E-MTAB-6653 (Lambrechts, D., 2018), E-MTAB-6149 (Lambrechts, D., 2018), GSE131907_Lung_Cancer (Kim, N., 2020). The open-access data downloaded from these researches concluded different lung diseases such as Idiopathic pulmonary fibrosis (IPF), chronic obstructive pulmonary disease (COPD), Systemic sclerosis (SSC), lung squamous carcinoma (LUSC), lung adenocarcinoma (LUAD) and large cell cancer (LCC). Those datasets are generated in 10x Genomics platform on V1 chemistry or on V2 chemistry, and we eliminated samples of other organs in each dataset. Eventually, the sc-RNAseq data of lung tissues analyzed was from overall 149 patients. The information of patients was shown in Table S2, including samples from 20 healthy controls, 15 chronic obstructive pulmonary disease (COPD) patients, 15 idiopathic pulmonary fibrosis (IPF) patients, 8 systemic sclerosis (SSC) patients, 65 large cell lung cancer (LCC) patients, 15 lung adenocarcinoma (LUAD) patients and 11 para cancer tissues.

**Sc-RNA seq data processing** Due to the multiple forms of supplied data in the public database, we transfer all raw data in form of fastq files into gene expression matrix using Cell Ranger toolkit (version 3.0.0, 10x Genomics) with default settings (https:// support. 10xgenomics. Com / single – cell – gene – expression / software/pipelines/latest/what-is-cell-ranger). For subsequent sc-RNA seq data analysis, single-cell transcriptome sequencing data were processed and converted to Seurat object using the Seurat R package (version 3.0). And then the standard workflow of quality control was performed. The Seurat object was generated according to the criteria that each gene was expressed by at least 3 cells and that at least 200 genes were expressed each cell. Before the downstream data processing, we further filter out the cells with low quality based on the following criteria: (1) unique gene count per cell >200 or <0.05 (2) mitochondrial genes > 10% or 20% in each sample based on the distribution of mitochondrial genes which potentially accounts to doublet cells.

**Sample integration and cluster analysis** As we have the dataset of 613952 cells and 16490 genes in total after quality control, we use harmony[7] and symphony[8] to identify cell type (Table S3). Thanks to an excellent work of lung cell atlas[9] of Travaglini et al, we have RNA expression of 58 types of lung cells. Single-cell reference mapping tools like symphony enables us annotate unlabeled dataset automatically. After deduplication and optimization, here we use three healthy lung scRNAseq dataset are integrated by harmony to annotate our collected dataset through symphony. Finally, a total of 57 cell subtypes and 4 main types were identified including 15 in epithelial cells, 9 in endothelial cells, 9 in stromal cells and 25 in immune cells. The detailed marker gene list was presented in the Table S1. For the quantitation, average gene expressions of cell markers in each panel of those cell types were calculated to compare the specificity of these 57 marker panels in different types of cells under various pathological conditions. The results were visualized by box plot.

**The calculation of overlap express rate (OER) of each marker gene panel in 57 types/subtypes of pulmonary single cells**

Take the evaluation of cell identity marker gene panel (ciMGP) in airway smooth muscle cell as an example in the following figures. The red box indicated the average mRNA expression of the target cell (airway smooth muscle cell)’s ciMGP (the sum of the mRNA level of gene ATAC, ACTA2, TAGLN, CNN1, MYH11, DES and KCNA5) to be tested. In the box plot, the top line and bottom line respectively indicated the upper quartile and lower quartile value. The values representing the difference between the upper quartile and lower quartile was divided evenly into four parts with the blue frames as seen in the following figure. If the upper quartile value in the box of another cell type lower than the 25% of difference value which was considered as the negative events (as can be seen of fibromyocyte in the following figure). While if the upper quartile value in the box of another cell is higher than the 50% of the difference value which was considered as the positive events (as can be seen of vascular smooth muscle cell in the following figure). The detailed calculation formula of the overlap expression rate (OER) was from the following equation: overlapped marker cell subset number/total cell subset number *100%. Cell-specific panel with <5% OER; cell-associated panel with 5%-10% OER; cell-reference panel with more than 10% OER.


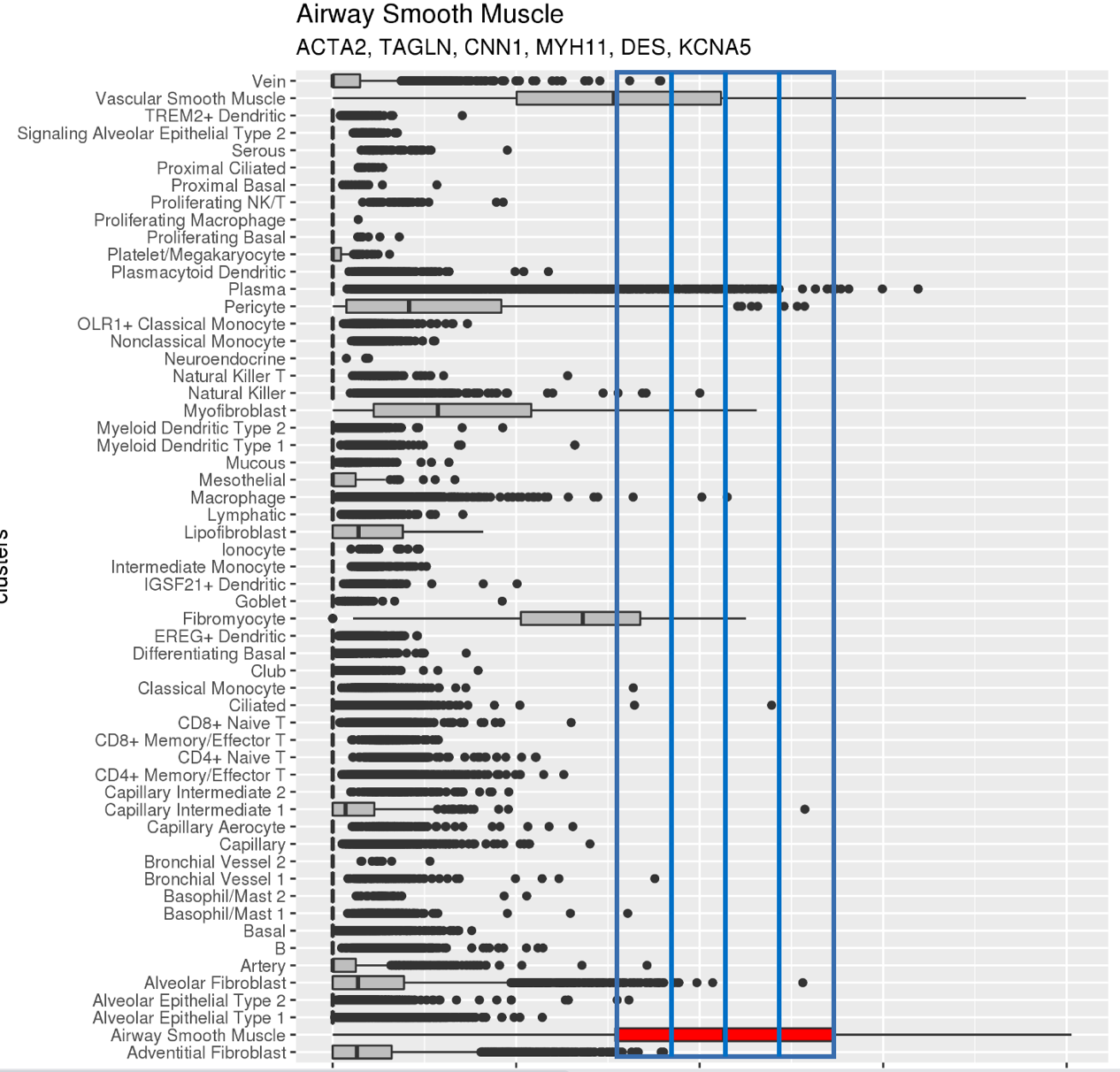

Supplement: Supplementary file 2 — Supporting Information [file CTM2-12-e1132-s002.docx]
