# Supplementary material for: Evaluation of pulmonary single‐cell identity specificity in scRNA‐seq analysis
Source: Clin Transl Med. 2022 Dec 10;12(12):e1132. doi: 10.1002/ctm2.1132 (PMC9736794; doi:10.1002/ctm2.1132)
Supplement: Supplementary file 3 — Supporting Information [file CTM2-12-e1132-s011.docx]

Supplement Table 1. The marker panel used in the single-cell RNA sequencing data of lung samples.

| **Main cell type** | **Cell type** | **Used markers** | **Number of marker gene** |
| --- | --- | --- | --- |
| Epithelial cell | Alveolar Epithelial Type 1 | HOPX, PDPN, CLIC5, AGER, CLDN18, EMP2 | 6 |
|  | Alveolar Epithelial Type 2 | SFTPB, SFTPC, SFTPD, ETV5, MUC1, WIF1, HHIP | 7 |
|  | Basal | KRT5, KRT14 | 2 |
|  | Ciliated | FOXJ1 | 1 |
|  | Club | SCGB1A1, SCGB3A2 | 2 |
|  | Differentiating Basal | KRT7, KRT13 | 2 |
|  | Goblet | MUC5AC, MUC5B | 2 |
|  | Mucous | MUC5B, MUC5AC | 2 |
|  | Neuroendocrine | ASCL1, CHGA | 2 |
|  | Proliferating Basal | MKI67 | 1 |
|  | Proximal Basal | KRT5, KRT14 | 2 |
|  | Proximal Ciliated | FOXJ1 | 1 |
|  | Signaling Alveolar Epithelial Type 2 | CP, SFTPC | 2 |
|  | Serous | LPO, LTF | 2 |
|  | Ionocyte | FOXI1, CFTR | 2 |
| Endothelial cell | Artery | CXCL12, DKK2, GJA5, SERPINE2 | 4 |
|  | Vein | PTGIS, CPE | 2 |
|  | Bronchial Vessel 1 | MYC, SPRY1 | 2 |
|  | Bronchial Vessel 2 | MYC | 1 |
|  | Capillary | IL7R, SLC6A4, FCN3 | 3 |
|  | Capillary Aerocyte | APLN, EDNRB, HPGD | 3 |
|  | Capillary Intermediate 1 | APLN, EDNRB, HPGD, IL1RL1 | 4 |
|  | Capillary Intermediate 2 | IL7R, SLC6A4, FCN3 | 3 |
|  | Lymphatic | TFF3, PDPN, CCL21, IGF1 | 4 |
| Stromal cell | Adventitial Fibroblast | SERPINF1, PI16, FBLN1, SCARA5 | 4 |
|  | Airway Smooth Muscle | ACTA2, TAGLN, CNN1, MYH11, DES, KCNA5 | 6 |
|  | Alveolar Fibroblast | SLC38A5, GDF10, GPC3, ITGA8 | 4 |
|  | Lipofibroblast | PLIN2, APOE | 2 |
|  | Myofibroblast | ACTA2, MYH11, ASPN, TYRP1 | 4 |
|  | Pericyte | COX4I2, HIGD1B, GJA4 | 3 |
|  | Mesothelial | MSLN, KRT19, UPK3B | 3 |
|  | Fibromyocyte | ASPN, FGF18, ACTA2, TAGLN, CNN1 | 5 |
|  | Vascular Smooth Muscle | ACTA2, TAGLN, CNN1, MYH11 | 4 |
| Immune cell | B | CD79A, MS4A1, CD19 | 3 |
|  | Basophil/Mast 1 | CPA3, MS4A2, RGS13, GATA2, KIT | 5 |
|  | Basophil/Mast 2 | CPA3, MS4A2, RGS13, GATA2, KIT | 5 |
|  | CD4+ Memory/Effector T | CD3E, CD4, LTB, COTL1, LDHB | 5 |
|  | CD4+ Naive T | CD3E, CD4, LTB, LDHB, CCR7, LEF1 | 6 |
|  | CD8+ Memory/Effector T | CD3E, GZMK, KLRB1, IL7R, DUSP2 | 6 |
|  | CD8+ Naive T | CD3E, GZMH, GZMB | 3 |
|  | Classical Monocyte | LGALS2, CD14, NRG1, S100A8, S100A9, S100A12, CD14 | 7 |
|  | EREG+ Dendritic | GPR183, EREG, NAMPT, CD14 | 4 |
|  | IGSF21+ Dendritic | GPR183, IGSF21, CD14 | 3 |
|  | Intermediate Monocyte | CD14, S100A8 | 2 |
|  | Macrophage | MARCO, MRC1, MSR1 | 3 |
|  | Myeloid Dendritic Type 1 | FCER1A, CD1C, LAMP3, CLEC9A | 4 |
|  | Myeloid Dendritic Type 2 | FCER1A, CD1C, PAK1, PKIB | 4 |
|  | Natural Killer | FCER1G, TYROBP, GZMB, CHST2, HOPX | 5 |
|  | Natural Killer T | CD3E, FCER1G, TYROBP | 3 |
|  | Nonclassical Monocyte | CDKN1C, CD79B, LYPD2, CHST2, IFITM2, HES4 | 4 |
|  | OLR1+ Classical Monocyte | OLR1 | 1 |
|  | Plasma | CD79A, CD27 | 2 |
|  | Plasmacytoid Dendritic | LILRA4, SCT, LRRC26, GZMB | 4 |
|  | Platelet/Megakaryocyte | CD14, CD33 | 2 |
|  | Proliferating Macrophage | MKI67, TOP2A, MARCO, MRC1, MSR1, CD74 | 6 |
|  | Proliferating NK/T | MKI67, TOP2A, CD3E, FCER1G | 4 |
|  | TREM2+ Dendritic | GPR183, TREM2, CD14 | 3 |
