# Supplementary material for: Evaluation of pulmonary single‐cell identity specificity in scRNA‐seq analysis
Source: Clin Transl Med. 2022 Dec 10;12(12):e1132. doi: 10.1002/ctm2.1132 (PMC9736794; doi:10.1002/ctm2.1132)
Supplement: Supplementary file 4 — Supporting Information [file CTM2-12-e1132-s012.docx]

Supplement Table 2. The information of samples.

| Published paper | Sample number | Average cells for each patient in this paper |
| --- | --- | --- |
| GSE136831 | 78 | 4011 |
| GSE128169 | 13 | 5099 |
| GSE128033 | 18 | 3972 |
| E_MTAB_6653 | 6 | 2855 |
| E-MTAB-6149 | 13 | 3528 |
| GSE131907_Lung_Cancer | 26 | 3854 |
