# Supplementary material for: Evaluation of pulmonary single‐cell identity specificity in scRNA‐seq analysis
Source: Clin Transl Med. 2022 Dec 10;12(12):e1132. doi: 10.1002/ctm2.1132 (PMC9736794; doi:10.1002/ctm2.1132)
Supplement: Supplementary file 5 — Supporting Information [file CTM2-12-e1132-s013.docx]

Supplemental Table 3. The information of diseases and cell number

| Disease | Cell # in endothelial | Cell # in epithelial | Cell # in  immune | Cell # in stromal |
| --- | --- | --- | --- | --- |
| NOR | 8472 | 17234 | 141045 | 6564 |
| PC-NOR | 2044 | 2248 | 13700 | 561 |
| COPD | 1344 | 3550 | 56664 | 1437 |
| IPF | 9343 | 21252 | 129993 | 10313 |
| SSC | 4526 | 7483 | 16414 | 3453 |
| LCC | 360 | 2573 | 3436 | 450 |
| LUAD | 1317 | 16224 | 47255 | 2632 |
