# Supplementary material for: Evaluation of pulmonary single‐cell identity specificity in scRNA‐seq analysis
Source: Clin Transl Med. 2022 Dec 10;12(12):e1132. doi: 10.1002/ctm2.1132 (PMC9736794; doi:10.1002/ctm2.1132)
Supplement: Supplementary file 6 — Supporting Information [file CTM2-12-e1132-s001.docx]

Supplemental Table 1. “Overlap expression cell subset rate” of cell subset-specific, cell subset-associated, cell subset-reference mark gen panels in total, normal (Norm), and para-cancer human lung tissues.

| Cell subset-specific(n=22) | | | | Cell subset-associated(n=8) | | | | Cell subset-reference(n=21) | | | | Negative expression(n=6) | | | |  |
| --- | --- | --- | --- | --- | --- | --- | --- | --- | --- | --- | --- | --- | --- | --- | --- | --- |
| Subset | Total | Norm | Para | Subset | Total | Norm | Para | Subset | Total | Norm | Para | Subset | Total | Norm | Para |  |
|  |  |  |  |  |  |  |  |  |  |  |  |  |  |  |  |  |
| Adventitial fibroblast | 0.0 | 0.0 | 0.0 | Classical monocyte | 5.4 | 3.6 | 1.8 | Bronchial vessel endothelia 1 | 33.9 | 26.8 | ND | Bronchial Vessel endothelia 2 | ND | ND | ND |  |
| Airway smooth muscle cell | 1.8 | 3.6 | 0.0 | Fibromyocyte | 5.4 | 7.1 | ND | Capillary Intermediate endothelia 2 | 30.4 | 23.2 | 28.6 | Ionocyte | ND | ND | ND |  |
| Alveolar epithelial type 1 | 0.0 | 0.0 | 0.0 | Macrophage | 5.4 | 7.1 | 0.0 | CD4+ Memory /Effector T cell | 28.6 | 35.7 | 21.4 | Plasma cell | ND | ND | ND |  |
| Alveolar epithelial type 2 | 1.8 | 1.8 | 1.8 | Myeloid dendritic type 1 | 8.9 | 8.9 | 10.7 | CD4+ Naive T cell | 73.2 | 76.8 | 60.7 | Plasmacytoid dendritic | ND | ND | 12.5 |  |
| Alveolar fibroblast | 1.8 | 3.6 | 0.0 | Myofibroblast | 7.1 | 8.9 | 3.6 | CD8+ Memory /Effector T cell | 10.7 | 10.7 | 10.7 | Proliferating basal epithelia | ND | ND | ND |  |
| Artery endothelia | 0.0 | 0.0 | 0.0 | CD8+ Naive T cell | 8.9 | 5.4 | 8.9 | Club epithelia | 10.7 | 5.4 | 1.8 | Serous epithelia | ND | ND | 0.0 |  |
| B cell | 0.0 | 0.0 | 0.0 | Capillary endothelia | 8.9 | 7.1 | 5.4 | Differentiating Basal epithelia | 14.3 | 19.6 | ND |  |  |  |  |  |
| Basal epithelia | 1.8 | 3.6 | 0.0 | Capillary Intermediate endothelia 1 | 8.9 | 5.4 | 3.6 | EREG+ Dendritic | 12.5 | 14.3 | 10.7 |  |  |  |  |  |
| Basophil/mast 1 | 1.8 | 3.6 | 0.0 |  |  |  |  | IGSF21+ Dendritic | 16.1 | 16.1 | 10.7 |  |  |  |  |  |
| Basophil/mast 2 | 1.8 | 1.8 | 1.8 |  |  |  |  | Intermediate monocyte | 25.0 | 26.8 | 28.6 |  |  |  |  |  |
| Goblet epithelia | 3.6 | 0 | 1.8 |  |  |  |  | Lipofibroblast | 78.6 | ND | ND |  |  |  |  |  |
| Capillary aerocyte | 0.0 | 1.8 | 1.8 |  |  |  |  | Myeloid dendritic type 2 | 35.7 | 23.2 | 21.4 |  |  |  |  |  |
| Ciliated epithelia | 3.6 | 1.8 | 0.0 |  |  |  |  | Natural Killer cell | 35.7 | 35.7 | 32.1 |  |  |  |  |  |
| Lymphatic endothelia | 0.0 | 0.0 | 0.0 |  |  |  |  | Natural Killer T cell | 44.6 | 37.5 | 35.7 |  |  |  |  |  |
| Mesothelial cell | 1.8 | 0.0 | 1.8 |  |  |  |  | Non-classical monocyte | 94.6 | 85.7 | 67.9 |  |  |  |  |  |
| Mucous epithelia | 3.6 | 3.6 | 3.6 |  |  |  |  | OLR1+ classical monocyte | 21.4 | 21.4 | 14.3 |  |  |  |  |  |
| Neuroendocrine epithelia | 0.0 | 0.0 | ND |  |  |  |  | Platelet/Megakaryocyte | 26.8 | 28.6 | ND |  |  |  |  |  |
| Pericyte cell | 0.0 | 0.0 | 0.0 |  |  |  |  | Proliferating macrophage | 10.7 | 8.9 | ND |  |  |  |  |  |
| Proximal basal epithelia | 1.8 | 5.4 | ND |  |  |  |  | Proliferating NK/T cell | 46.4 | 58.9 | 50 |  |  |  |  |  |
| Proximal ciliated epithelia | 1.8 | 1.8 | ND |  |  |  |  | TREM2+ dendritic | 14.3 | 23.2 | 25.0 |  |  |  |  |  |
| Signaling alveolar epithelial type 2 | 1.8 | 1.8 | 1.8 |  |  |  |  | Vein endothelia | 21.5 | 8.9 | 1.7 |  |  |  |  |  |
| Vascular smooth muscle cell | 3.6 | 5.4 | 3.6 |  |  |  |  |  |  |  |  |  |  |  |  |  |
