# Supplementary material for: Evaluation of pulmonary single‐cell identity specificity in scRNA‐seq analysis
Source: Clin Transl Med. 2022 Dec 10;12(12):e1132. doi: 10.1002/ctm2.1132 (PMC9736794; doi:10.1002/ctm2.1132)
Supplement: Supplementary file 7 — Supporting Information [file CTM2-12-e1132-s008.docx]

Supplemental Table 2. “Overlap expression cell subset rate” of human lung tissues harvested from patients with lung adenocarcinoma (LUAD), large cell cancer (LCC), idiopathic pulmonary fibrosis (IPF), chronic obstructive pulmonary disease (COPD), and systemic sclerosis (SSC), as compared with reference values which were the mean value of mark gen panels in total, normal (Norm), and para-cancer human lung tissues.

| Cell subsets | Refs | LCC | LUAD | IPF | COPD | SSC |
| --- | --- | --- | --- | --- | --- | --- |
| Adventitial fibroblast | 0.0 | 3.6 | 3.6 | 1.8 | 0.0 | 1.8 |
| Airway smooth muscle cell | 1.8 | 1.8 | 0.0 | 1.8 | 1.8 | 1.8 |
| Alveolar epithelial type 1 | 0.0 | 41.1 | 5.4 | 1.8 | 0.0 | 0.0 |
| Alveolar epithelial type 2 | 1.8 | 5.4 | 1.8 | 1.8 | 1.8 | 1.8 |
| Alveolar fibroblast | 1.8 | 1.8 | 1.8 | 1.8 | 0.0 | 1.8 |
| Artery endothelia | 0.0 | 3.6 | 0.0 | 0.0 | 0.0 | 1.8 |
| B cell | 0.0 | 1.8 | 1.8 | 0.0 | 0.0 | 0.0 |
| Basal epithelia | 1.8 | 0.0 | ND | 3.6 | 3.6 | 3.6 |
| Basophil/mast 1 | 1.8 | ND | ND | 3.6 | 3.6 | 3.6 |
| Basophil/mast 2 | 1.8 | 1.8 | 1.8 | 1.8 | 1.8 | 1.8 |
| Bronchial vessel endothelia 1 | 33.9 | 3.6 | 25 | 5.4 | 30.4 | 16.1 |
| Bronchial Vessel endothelia 2 | 0.0 | ND | ND | ND | ND | ND |
| Capillary endothelia | 8.9 | 14.3 | 14.3 | 23.2 | 7.1 | 3.6 |
| Capillary aerocyte | 0 | 0.0 | 0.0 | 0.0 | 1.8 | 1.8 |
| Capillary Intermediate endothelia 1 | 8.9 | ND | 5.4 | 7.1 | 5.4 | 12.5 |
| Capillary Intermediate endothelia 2 | 30.4 | 21.4 | 26.8 | 37.5 | 23.2 | 30.4 |
| CD4+ Memory/Effector T cell | 28.6 | 21.4 | 12.5 | 41.1 | 85.7 | 25.0 |
| CD4+ Naive T cell | 73.2 | 14.3 | 14.3 | 91.1 | 76.8 | 62.5 |
| CD8+ Memory/Effector T cell | 10.7 | 10.7 | 10.7 | 10.7 | 10.7 | 8.9 |
| CD8+ Naive T cell | 8.9 | 8.9 | 8.9 | 7.1 | 7.1 | 10.7 |
| Ciliated epithelia | 3.6 | 0.0 | 0.0 | 3.6 | 3.6 | 3.6 |
| Classical monocyte | 5.4 | 3.6 | 3.6 | 5.4 | 1.8 | 1.8 |
| Club epithelia | 10.7 | 67.9 | 12.5 | 7.1 | 5.4 | 7.1 |
| Differentiating Basal epithelia | 14.3 | 17.9 | 14.3 | 17.9 | 23.2 | 21.4 |
| EREG+ Dendritic | 12.5 | 10.7 | 7.1 | 14.3 | 10.7 | 10.7 |
| Fibromyocyte | 5.4 | 5.4 | 5.4 | 5.4 | 12.5 | 7.1 |
| Goblet epithelia | 3.6 | 5.4 | 3.6 | 3.6 | 1.8 | 3.6 |
| IGSF21+ Dendritic | 16.1 | 10.7 | 14.3 | 16.1 | 14.3 | 16.1 |
| Intermediate monocyte | 25 | 14.3 | 16.1 | 21.4 | 25.0 | 19.6 |
| Ionocyte | 0.0 | ND | ND | ND | ND | ND |
| Lipofibroblast | 78.6 | ND | 89.3 | 96.4 | ND | 42.9 |
| Lymphatic endothelia | 0.0 | 12.5 | 0.0 | 0.0 | 0.0 | 0.0 |
| Macrophage | 5.4 | 14.3 | 5.4 | 7.1 | 3.6 | 8.9 |
| Mesothelial cell | 1.8 | 21.4 | 12.5 | 0.0 | 0.0 | ND |
| Mucous epithelia | 3.6 | 5.4 | 3.6 | 3.6 | 1.8 | 3.6 |
| Myeloid dendritic type 1 | 8.9 | 8.9 | 7.1 | 8.9 | 10.7 | 8.9 |
| Myeloid dendritic type 2 | 35.7 | 33.9 | 30.4 | 37.5 | 30.4 | 16.1 |
| Myofibroblast | 7.1 | 7.1 | 8.9 | 10.7 | 12.5 | 7.1 |
| Natural Killer cell | 35.7 | 57.1 | 33.9 | 35.7 | 64.3 | 26.8 |
| Natural Killer T cell | 44.6 | 32.1 | 35.7 | 44.6 | 39.3 | 41.1 |
| Neuroendocrine epithelia | 0.0 | ND | ND | ND | 0.0 | ND |
| Non-classical monocyte | 94.6 | 23.2 | 94.6 | 96.4 | 42.9 | 91.1 |
| OLR1+ classical monocyte | 21.4 | 5.4 | 16.1 | 23.2 | 17.9 | 21.4 |
| Pericyte cell | 0.0 | 1.8 | 0.0 | 0.0 | 0.0 | 1.8 |
| Plasma cell | 0.0 | ND | ND | ND | ND | ND |
| Plasmacytoid dendritic | 0 | ND | ND | ND | ND | ND |
| Platelet/Megakaryocyte | 26.8 | ND | 19.6 | 25.0 | ND | 26.8 |
| Proliferating basal epithelia | 0.0 | ND | ND | ND | ND | ND |
| Proliferating macrophage | 10.7 | ND | ND | 8.9 | ND | 16.1 |
| Proliferating NK/T cell | 51.2 | 37.5 | 41.1 | 44.6 | 53.6 | ND |
| Proximal basal epithelia | 1.8 | ND | ND | 3.6 | 3.6 | 3.6 |
| Proximal ciliated epithelia | 1.8 | ND | ND | 1.8 | 3.6 | 3.6 |
| Serous epithelia | 0.0 | 3.6 | 7.1 | ND | 7.1 | ND |
| Signaling alveolar epithelial type 2 | 1.8 | 0.0 | 48.2 | 7.1 | 1.8 | 1.8 |
| TREM2+ dendritic | 14.3 | 14.3 | 16.1 | 17.9 | 25.0 | 17.9 |
| Vascular smooth muscle cell | 3.6 | 1.8 | 3.6 | 3.6 | 1.8 | 3.6 |
| Vein endothelia | 21.4 | 35.7 | 16.1 | 17.9 | 17.9 | 14.3 |
