# Supplementary material for: Evaluation of pulmonary single‐cell identity specificity in scRNA‐seq analysis
Source: Clin Transl Med. 2022 Dec 10;12(12):e1132. doi: 10.1002/ctm2.1132 (PMC9736794; doi:10.1002/ctm2.1132)
Supplement: Supplementary file 8 — Supporting Information [file CTM2-12-e1132-s005.docx]

Supplemental Table 6.Overlap expression cell subset of each cell subset marker gene panel of human lung tissues harvested from patients with lung adenocarcinoma (LUAD), large cell cancer (LCC), idiopathic pulmonary fibrosis (IPF), chronic obstructive pulmonary disease (COPD), and systemic sclerosis (SSC) total, normal (Norm), and para-cancer human lung tissues.

| **Cell subset** | **Gene panel** | **Total** | **Normal** | **Para-cancer** | **LCC** | **LUAD** | **IPF** | **COPD** | **SSC** |
| --- | --- | --- | --- | --- | --- | --- | --- | --- | --- |
| Basophil/Mast 2 | CPA3, MS4A2, RGS13, GATA2, KIT | Basophil/Mast 1 | Basophil/Mast 1 | Basophil/Mast 1 | Basophil/Mast 1 | Basophil/Mast 1 | Basophil/Mast 1 | Basophil/Mast 1 | Basophil/Mast 1 |
| Classical Monocyte | LGALS2, CD14, NRG1, S100A8, S100A9, S100A12, CD14 | OLR1+classic monocyte, Intermediate monocyte, EREG+ dendritic | OLR1+classic monocyte, EREG+ dendritic | OLR1+classic monocyte | OLR1+classic monocyte, Intermediate monocyte | OLR1+classic monocyte, Intermediate monocyte | OLR1+classic monocyte, Intermediate monocyte, EREG+ dendritic | OLR1+classic monocyte | OLR1+classic monocyte |
| EREG+ Dendritic | GPR183, EREG, NAMPT, CD14 | OLR1+classic monocyte,  Nonclassical monocyte, Myeloid dendritic type 2  Myeloid dendritic type 1, Intermediate monocyte  IGSF21+ dendritic, Classical monocyte | Plasmacytoid dendritic, OLR1+classic monocyte,  Nonclassical monocyte, Myeloid dendritic type 2  Myeloid dendritic type 1, Intermediate monocyte  IGSF21+ dendritic, Classical monocyte | OLR1+classic monocyte, Myeloid dendritic type 2  Myeloid dendritic type 1, Intermediate monocyte  IGSF21+ dendritic, Classical monocyte | OLR1+classic monocyte, Myeloid dendritic type 2,  Myeloid dendritic type 1, Intermediate monocyte  IGSF21+ dendritic, Classical monocyte | OLR1+classic monocyte, Myeloid dendritic type 1, IGSF21+ dendritic, Classical monocyte | Plasmacytoid dendritic, OLR1+classic monocyte,  Nonclassical monocyte, Myeloid dendritic type 2  Myeloid dendritic type 1, Intermediate monocyte  IGSF21+ dendritic, Classical monocyte | OLR1+classic monocyte, Myeloid dendritic type 2  Myeloid dendritic type 1, Intermediate monocyte  IGSF21+ dendritic, Classical monocyte | OLR1+classic monocyte, Myeloid dendritic type 2  Myeloid dendritic type 1, Intermediate monocyte  IGSF21+ dendritic, Classical monocyte |
| IGSF21+ Dendritic | GPR183, IGSF21, CD14 | TREM2+dendritic, Plasmacytoid dendritic ,OLR1+classic monocyte  Nonclassical monocyte, Myeloid dendritic type 2  Myeloid dendritic type 1, Intermediate monocyte, EREG+ dendritic, Classical monocyte | TREM2+dendritic, Plasmacytoid dendritic ,OLR1+classic monocyte,  Nonclassical monocyte, Myeloid dendritic type 2  Myeloid dendritic type 1, Intermediate monocyte, EREG+ dendritic, Classical monocyte | OLR1+classic monocyte, Myeloid dendritic type 2  Myeloid dendritic type 1, Intermediate monocyte, EREG+ dendritic, Classical monocyte | TREM2+dendritic, OLR1+classic monocyte, Nonclassical monocyte, Myeloid dendritic type 2  Myeloid dendritic type 1, Intermediate monocyte, EREG+ dendritic, Classical monocyte | TREM2+dendritic, Plasmacytoid dendritic , OLR1+classic monocyte,  Nonclassical monocyte, Myeloid dendritic type 2  Myeloid dendritic type 1, Intermediate monocyte, EREG+ dendritic, Classical monocyte | TREM2+dendritic, Plasmacytoid dendritic , OLR1+classic monocyte,  Nonclassical monocyte, Myeloid dendritic type 2  Myeloid dendritic type 1, Intermediate monocyte, EREG+ dendritic, Classical monocyte | Plasmacytoid dendritic , OLR1+classic monocyte, Myeloid dendritic type 2  Myeloid dendritic type 1, Intermediate monocyte, EREG+ dendritic, Classical monocyte,B cell | TREM2+dendritic, Plasmacytoid dendritic , OLR1+classic monocyte,  Nonclassical monocyte, Myeloid dendritic type 2  Myeloid dendritic type 1, Intermediate monocyte, EREG+ dendritic, Classical monocyte |
| Intermediate Monocyte | CD14, S100A8 | TREM2+dendritic, Proliferating macrophage, Platelet/Megakaryocyte,  Plasmacytoid dendritic,  Plasma cell, OLR1+classic monocyte,  Nonclassical monocyte, Myeloid dendritic type 2,  Myeloid dendritic type 1, Macrophage, IGSF21+ dendritic, EREG+ dendritic, Classical monocyte, Capillary aerocyte | TREM2+dendritic, Proliferating macrophage, Platelet/Megakaryocyte,  Plasmacytoid dendritic,  Plasma cell, OLR1+classic monocyte,  Nonclassical monocyte, Myeloid dendritic type 2,  Myeloid dendritic type 1, Macrophage, IGSF21+ dendritic, EREG+ dendritic, Classical monocyte, Capillary aerocyte,  Capillary endothelia | TREM2+dendritic, Serous epithelia, Plasma cell,  OLR1+classic monocyte,  Nonclassical monocyte, Myeloid dendritic type 2, Macrophage, IGSF21+ dendritic, EREG+ dendritic, Classical monocyte,  Capillary intermediate endothelia 2,  Capillary intermediate endothelia 1, Capillary endothelia,  Bronchial vessel endothelia 2,  Bronchial vessel endothelia 1, B cell | TREM2+dendritic, OLR1+classic monocyte,  Nonclassical monocyte, Myeloid dendritic type 2, Macrophage, IGSF21+ dendritic, EREG+ dendritic, Classical monocyte | TREM2+dendritic, Platelet/Megakaryocyte, OLR1+classic monocyte,  Nonclassical monocyte, Myeloid dendritic type 2, Macrophage, IGSF21+ dendritic, EREG+ dendritic, Classical monocyte | TREM2+dendritic, Proliferating macrophage, Platelet/Megakaryocyte,  Plasmacytoid dendritic,  Plasma cell, OLR1+classic monocyte,  Nonclassical monocyte, Myeloid dendritic type 2,  Myeloid dendritic type 1, Macrophage, IGSF21+ dendritic, EREG+ dendritic, Classical monocyte | TREM2+dendritic, Platelet/Megakaryocyte,  Plasmacytoid dendritic,  Plasma cell, OLR1+classic monocyte,  Nonclassical monocyte, Macrophage, IGSF21+ dendritic, EREG+ dendritic, Classical monocyte, Capillary intermediate endothelia 1. Capillary endothelia | TREM2+dendritic, OLR1+classic monocyte,  Nonclassical monocyte, Myeloid dendritic type 2, Macrophage, IGSF21+ dendritic, EREG+ dendritic, Classical monocyte, Capillary endothelia, Bronchial vessel endothelia 2 |
| Macrophage | MARCO, MRC1, MSR1 | TREM2+dendritic, Proliferating macrophage  ,Platelet/Megakaryocyte, | TREM2+dendritic, Proliferating macrophage  ,Platelet/Megakaryocyte, Plasma cell | 0 | TREM2+dendritic, OLR1+classic monocyte  Nonclassical monocyte, Myeloid dendritic type 2, Intermediate monocyte,  IGSF21+ dendritic, EREG+ dendritic, Classical monocyte | TREM2+dendritic, Myeloid dendritic type 2, IGSF21+ dendritic | TREM2+dendritic, Proliferating macrophage, Platelet/Megakaryocyte, Plasma cell | TREM2+dendritic, Plasma cell | TREM2+dendritic, Platelet/Megakaryocyte, Myeloid dendritic type 2, IGSF21+ dendritic, EREG+ dendritic |
| Myeloid Dendritic Type 1 | FCER1A, CD1C, LAMP3, CLEC9A | Signaling_AT2, Myeloid dendritic type 2, Basophil/Mast 2  Basophil/Mast 1,AT2 | Signaling_AT2, Myeloid dendritic type 2, Basophil/Mast 2  Basophil/Mast 1,AT2 | Signaling_AT2, Plasmacytoid dendritic  , Myeloid dendritic type 2, Basophil/Mast 2  Basophil/Mast 1,AT2 | Signaling_AT2, Myeloid dendritic type 2, EREG+ dendritic,  Basophil/Mast 1,AT2 | Myeloid dendritic type 2, Basophil/Mast 2  Basophil/Mast 1,AT2 | Signaling_AT2, Myeloid dendritic type 2, Basophil/Mast 2  Basophil/Mast 1,AT2 | Signaling_AT2, Myeloid dendritic type 2, IGSF21+ dendritic, EREG+ dendritic, Capillary intermediate endothelia 1, AT2 | Signaling_AT2, Intermediate monocyte, asophil/Mast 2  Basophil/Mast 1, AT2 |
| Myeloid Dendritic Type 2 | FCER1A, CD1C, PAK1, PKIB | TREM2+dendritic, Proximal ciliated epithelia  Proximal basal epithelia, Proliferating macrophage  Proliferating basal epithelia  Platelet/Megakaryocyte, Neuroendocrine epithelia, Myeloid dendritic type 1, Mucous epithelia, Ionocyte,  Intermediate monocyte,  IGSF21+ dendritic,  Goblet epithelia, EREG+ dendritic  Differentiating basal epithelia  Club epithelia, Ciliated epithelia, Basophil/Mast 2  Basophil/Mast 1  Basal epithelia | TREM2+dendritic, Proximal ciliated epithelia  Proximal basal epithelia, Platelet/Megakaryocyte, Myeloid dendritic type 1, Intermediate monocyte,  IGSF21+ dendritic,  Goblet epithelia, EREG+ dendritic  Differentiating basal epithelia,  Ciliated epithelia, Basophil/Mast 2  Basophil/Mast 1  Basal epithelia | TREM2+dendritic, Plasmacytoid dendritic, Nonclassical monocyte, Myeloid dendritic type 1, Intermediate monocyte  IGSF21+ dendritic  Goblet epithelia, EREG+ dendritic, Ciliated epithelia, Basophil/Mast 2  Basophil/Mast 1, Basal epithelia | TREM2+dendritic, Proximal basal epithelia, Proliferating basal epithelia,  Platelet/Megakaryocyte, Nonclassical monocyte, Myeloid dendritic type 1  Mucous epithelia  Mesothelial cell,  Ionocyte  Intermediate monocyte  IGSF21+ dendritic,  Goblet epithelia, EREG+ dendritic  Differentiating basal epithelia,  Club epithelia, Ciliated epithelia, Basophil/Mast 1,  Basal epithelia, AT2 | TREM2+dendritic, Proximal basal epithelia, Proliferating basal epithelia,  Platelet/Megakaryocyte, Nonclassical monocyte, Myeloid dendritic type 1  Mucous epithelia, Intermediate monocyte  IGSF21+ dendritic,  Goblet epithelia, EREG+ dendritic  Differentiating basal epithelia,  Club epithelia, Ciliated epithelia, Basophil/Mast 1,  Basal epithelia, AT2 | Proximal ciliated epithelia  Proximal basal epithelia, Proliferating basal epithelia  Platelet/Megakaryocyte  Plasmacytoid dendritic, Neuroendocrine epithelia, Myeloid dendritic type 1  Mucous epithelia,  Mesothelial cell, Ionocyte  Intermediate monocyte,  IGSF21+ dendritic,  Goblet epithelia, EREG+ dendritic  Differentiating basal epithelia,  Club epithelia, Ciliated epithelia, Basophil/Mast 2,  Basophil/Mast 1,  Basal epithelia | TREM2+dendritic, Proximal basal epithelia, Proliferating basal epithelia,  Platelet/Megakaryocyte, Nonclassical monocyte, Myeloid dendritic type 1  Mucous epithelia, Intermediate monocyte  IGSF21+ dendritic,  Goblet epithelia, EREG+ dendritic  Differentiating basal epithelia,  Club epithelia, Ciliated epithelia, Basophil/Mast 1,  Basal epithelia, AT2 | Proximal basal epithelia, Proliferating macrophage, Platelet/Megakaryocyte, Myeloid dendritic type 1, Intermediate monocyte, EREG+ dendritic, Ciliated epithelia, Basophil/Mast 2  Basophil/Mast 1, |
| Nonclassical Monocyte | CDKN1C, CD79B, LYPD2, CHST2, IFITM2, HES4 | Vein endothelia  Vascular smooth muscle cell  Signaling AT2  Serous epithelia  Proximal ciliated epithelia  Proximal basal epithelia  Proliferating NK/T cell  Proliferating macrophage  Proliferating basal epithelia  Platelet/Megakaryocyte  Plasmacytoid dendritic  Pericyte cell  OLR1+classic monocyte  Neuroendocrine epithelia  Natural killer T cell  Natural killer  Myofibroblast  Myeloid dendritic type 2  Myeloid dendritic type 1  Mucous epithelia  Mesothelial cell  Lymphatic endothelia  Lipofibroblast  Ionocyte  Intermediate monocyte  IGSF21+ dendritic  Goblet epithelia  Fibromyocyte  EREG+ dendritic  Differentiating basal epithelia  Club epithelia  Classical monocyte  Ciliated epithelia  CD8+ naïve T cell  CD8+ memory/effector T cell  CD4+ naïve T cell  CD4+ memory/effector T cell  Capillary intermediate endothelia 2  Capillary intermediate endothelia 1  Capillary aerocyte  Capillary endothelia  Bronchial vessel endothelia 2  Bronchial vessel endothelia 1  Basophil/Mast 2  Basophil/Mast 1  Basal epithelia  B cell  Artery endothelia  Alveolar fibroblast  Alveolar epithelial type2  Alveolar epithelial type1  Airway smooth muscle cell  Adventitial fibroblast | Vein endothelia  Vascular smooth muscle cell  TREM2+dendritic  Signaling AT2  Serous epithelia  Proximal ciliated epithelia  Proximal basal epithelia  Proliferating NK/T cell  Proliferating basal epithelia  Platelet/Megakaryocyte  Plasmacytoid dendritic  Plasma cell  Pericyte cell  OLR1+classic monocyte  Natural killer T cell  Natural killer  Myofibroblast  Myeloid dendritic type 2  Myeloid dendritic type 1  Mucous epithelia  Mesothelial cell  Lymphatic endothelia  Intermediate monocyte  IGSF21+ dendritic  Goblet epithelia  Fibromyocyte  EREG+ dendritic  Differentiating basal epithelia  Club epithelia  Classical monocyte  Ciliated epithelia  CD8+ naïve T cell  CD8+ memory/effector T cell  CD4+ naïve T cell  CD4+ memory/effector T cell  Capillary intermediate endothelia 2  Capillary intermediate endothelia 1  Capillary aerocyte  Capillary endothelia  Bronchial vessel endothelia 2  Bronchial vessel endothelia 1  Basophil/Mast 2  Basophil/Mast 1  Basal epithelia  B cell  Artery endothelia  Alveolar fibroblast  Alveolar epithelial type2  Alveolar epithelial type1  Airway smooth muscle cell  Adventitial fibroblast | Vein endothelia  Vascular smooth muscle cell  Signaling AT2  Serous epithelia  Proximal ciliated epithelia  Proximal basal epithelia  Proliferating NK/T cell  Proliferating macrophage  Proliferating basal epithelia  Plasmacytoid dendritic  Pericyte cell  OLR1+classic monocyte  Neuroendocrine epithelia  Natural killer T cell  Natural killer  Myofibroblast  Myeloid dendritic type 1  Mesothelial cell  Lymphatic endothelia  Lipofibroblast  Ionocyte  Intermediate monocyte  IGSF21+ dendritic  Fibromyocyte  EREG+ dendritic  Differentiating basal epithelia  Club epithelia  Classical monocyte  Ciliated epithelia  CD8+ naïve T cell  CD8+ memory/effector T cell  CD4+ naïve T cell  CD4+ memory/effector T cell  Capillary intermediate endothelia 2  Capillary intermediate endothelia 1  Capillary aerocyte  Capillary endothelia  Bronchial vessel endothelia 2  Bronchial vessel endothelia 1  Basophil/Mast 2  Basophil/Mast 1  B cell  Artery endothelia  Alveolar fibroblast  Alveolar epithelial type2  Alveolar epithelial type1  Airway smooth muscle cell  Adventitial fibroblast | Vein endothelia,  Vascular smooth muscle cell, Lymphatic endothelia, Intermediate monocyte, Fibromyocyte, Capillary intermediate endothelia 2, Capillary aerocyte  Capillary endothelia, Bronchial vessel endothelia 1, Basophil/Mast 1,  B cell,  Artery endothelia | Vein endothelia  Vascular smooth muscle cell  TREM2+dendritic  Signaling AT2  Serous epithelia  Proximal ciliated epithelia  Proximal basal epithelia  Proliferating NK/T cell  Proliferating basal epithelia  Platelet/Megakaryocyte  Plasmacytoid dendritic  Plasma cell  Pericyte cell  OLR1+classic monocyte  Neuroendocrine epithelia  Natural killer T cell  Natural killer  Myofibroblast  Myeloid dendritic type 2  Myeloid dendritic type 1  Mucous epithelia  Mesothelial cell  Macrophage  Lymphatic endothelia  Lipofibroblast  Ionocyte  Intermediate monocyte  IGSF21+ dendritic  Goblet epithelia  Fibromyocyte  EREG+ dendritic  Differentiating basal epithelia  Club epithelia  Classical monocyte  Ciliated epithelia  CD8+ naïve T cell  CD8+ memory/effector T cell  CD4+ naïve T cell  CD4+ memory/effector T cell  Capillary intermediate endothelia 2  Capillary intermediate endothelia 1  Capillary aerocyte  Capillary endothelia  Bronchial vessel endothelia 1  Basophil/Mast 2  Basophil/Mast 1  Basal epithelia  B cell  Artery endothelia  Alveolar fibroblast  Alveolar epithelial type2  Alveolar epithelial type1  Airway smooth muscle cell  Adventitial fibroblast | Vein endothelia  Vascular smooth muscle cell  TREM2+dendritic  Signaling AT2  Serous epithelia  Proximal ciliated epithelia  Proximal basal epithelia  Proliferating NK/T cell  Proliferating macrophage  Proliferating basal epithelia  Platelet/Megakaryocyte  Plasmacytoid dendritic  Plasma cell  Pericyte cell  OLR1+classic monocyte  Neuroendocrine epithelia  Natural killer T cell  Natural killer  Myofibroblast  Myeloid dendritic type 2  Myeloid dendritic type 1  Mucous epithelia  Mesothelial cell  Lymphatic endothelia  Lipofibroblast  Ionocyte  Intermediate monocyte  IGSF21+ dendritic  Goblet epithelia  Fibromyocyte  EREG+ dendritic  Differentiating basal epithelia  Club epithelia  Classical monocyte  Ciliated epithelia  CD8+ naïve T cell  CD8+ memory/effector T cell  CD4+ naïve T cell  CD4+ memory/effector T cell  Capillary intermediate endothelia 2  Capillary intermediate endothelia 1  Capillary aerocyte  Capillary endothelia  Bronchial vessel endothelia 2  Bronchial vessel endothelia 1  Basophil/Mast 2  Basophil/Mast 1  Basal epithelia  B cell  Artery endothelia  Alveolar fibroblast  Alveolar epithelial type2  Alveolar epithelial type1  Airway smooth muscle cell  Adventitial fibroblast | Vein endothelia,  Vascular smooth muscle cell, Pericyte cell, Natural killer  Myofibroblast, Mesothelial cell, Lymphatic endothelia,  Intermediate monocyte, Goblet epithelia, Classical monocyte, CD8+ naïve T cell  CD8+ memory/effector T cell, Capillary intermediate endothelia 2  Capillary intermediate endothelia 1  Capillary aerocyte  Capillary endothelia  Bronchial vessel endothelia 2  Bronchial vessel endothelia 1, Basal epithelia, Artery endothelia, Alveolar fibroblast, Airway smooth muscle cell,  Adventitial fibroblast | Vein endothelia  Vascular smooth muscle cell  TREM2+dendritic  Signaling AT2  Serous epithelia  Proximal ciliated epithelia  Proximal basal epithelia  Proliferating NK/T cell  Proliferating macrophage  Proliferating basal epithelia  Platelet/Megakaryocyte  Plasmacytoid dendritic  Pericyte cell  OLR1+classic monocyte  Natural killer T cell  Natural killer  Myofibroblast  Myeloid dendritic type 2  Myeloid dendritic type 1  Mucous epithelia  Mesothelial cell  Macrophage  Lymphatic endothelia  Lipofibroblast  Intermediate monocyte  IGSF21+ dendritic  Goblet epithelia  Fibromyocyte  EREG+ dendritic  Differentiating basal epithelia  Club epithelia  Classical monocyte  Ciliated epithelia  CD8+ naïve T cell  CD8+ memory/effector T cell  CD4+ naïve T cell  CD4+ memory/effector T cell  Capillary intermediate endothelia 2  Capillary intermediate endothelia 1  Capillary aerocyte  Capillary endothelia  Bronchial vessel endothelia 2  Bronchial vessel endothelia 1  Basophil/Mast 2  Basophil/Mast 1  Basal epithelia  B cell  Artery endothelia  Alveolar fibroblast  Alveolar epithelial type2  Alveolar epithelial type1  Airway smooth muscle cell  Adventitial fibroblast |
| OLR1+ Classical Monocyte | OLR1 | TREM2+dendritic, Proliferating macrophage, Platelet/Megakaryocyte, Plasma cell, Nonclassical monocyte, Myeloid dendritic type 2,  Myeloid dendritic type 1, Macrophage  , Intermediate monocyte  IGSF21+ dendritic, EREG+ dendritic, Classical monocyte | TREM2+dendritic, Proliferating macrophage, Platelet/Megakaryocyte, Plasma cell, Nonclassical monocyte, Myeloid dendritic type 2,  Myeloid dendritic type 1, Macrophage  , Intermediate monocyte  IGSF21+ dendritic, EREG+ dendritic, Classical monocyte | TREM2+dendritic, Myeloid dendritic type 2,  Myeloid dendritic type 1, Macrophage, Intermediate monocyte  IGSF21+ dendritic, EREG+ dendritic, Classical monocyte | TREM2+dendritic, Myeloid dendritic type 2, EREG+ dendritic | TREM2+dendritic, Nonclassical monocyte, Myeloid dendritic type 2,  Myeloid dendritic type 1, Macrophage, Intermediate monocyte  IGSF21+ dendritic, EREG+ dendritic, Classical monocyte | TREM2+dendritic, Proliferating macrophage, Platelet/Megakaryocyte  Plasmacytoid dendritic  Plasma cell, Nonclassical monocyte, Myeloid dendritic type 2,  Myeloid dendritic type 1, Macrophage, Intermediate monocyte  IGSF21+ dendritic, EREG+ dendritic, Classical monocyte | TREM2+dendritic, Platelet/Megakaryocyte, Plasma cell, Myeloid dendritic type 2,  Myeloid dendritic type 1, Macrophage, Intermediate monocyte  IGSF21+ dendritic, EREG+ dendritic, Classical monocyte | TREM2+dendritic, Proliferating macrophage, Platelet/Megakaryocyte, Plasma cell, Nonclassical monocyte, Myeloid dendritic type 2,  Myeloid dendritic type 1, Macrophage  , Intermediate monocyte  IGSF21+ dendritic, EREG+ dendritic, Classical monocyte |
| Plasmacytoid Dendritic | LILRA4, SCT, LRRC26, GZMB | ND | ND | Proliferating NK/T cell, Natural killer T cell  Natural killer, Goblet epithelia, CD8+ naïve T cell  CD8+ memory/effector T cell, CD4+ memory/effector T cell | ND | ND | ND | ND | ND |
| Platelet/Megakaryocyte | CD14, CD33 | TREM2+dendritic, Proliferating macrophage, OLR1+classic monocyte  Nonclassical monocyte, Myeloid dendritic type 2  Myeloid dendritic type 1, Macrophage, Intermediate monocyte,  IGSF21+ dendritic, EREG+ dendritic, Classical monocyte, Capillary intermediate endothelia 1  Capillary aerocyte  Capillary endothelia  Bronchial vessel endothelia 2 | TREM2+dendritic, Proliferating macrophage, Plasmacytoid dendritic, OLR1+classic monocyte  Nonclassical monocyte,  Myeloid dendritic type 2  Myeloid dendritic type 1, Macrophage, Intermediate monocyte,  IGSF21+ dendritic, EREG+ dendritic, Classical monocyte, Capillary intermediate endothelia 1,  Capillary aerocyte  Capillary endothelia,  Bronchial vessel endothelia 2 | ND | ND | TREM2+dendritic, OLR1+classic monocyte  Nonclassical monocyte, Myeloid dendritic type 2  Myeloid dendritic type 1, Macrophage,  Intermediate monocyte,  IGSF21+ dendritic, EREG+ dendritic, Classical monocyte, Basophil/Mast 1 | TREM2+dendritic, Proliferating macrophage, OLR1+classic monocyte  Nonclassical monocyte  Neuroendocrine epithelia, Myeloid dendritic type 2  Myeloid dendritic type 1, Macrophage, Intermediate monocyte,  IGSF21+ dendritic, EREG+ dendritic, Classical monocyte, Capillary intermediate endothelia 1,  Capillary aerocyte, | ND | TREM2+dendritic, Proximal ciliated epithelia, OLR1+classic monocyte  Nonclassical monocyte, Myeloid dendritic type 2  Myeloid dendritic type 1, Macrophage, Intermediate monocyte,  IGSF21+ dendritic, Capillary intermediate endothelia 1  Capillary aerocyte  Capillary endothelia,  Bronchial vessel endothelia 2 |
| Proliferating Macrophage | MKI67, TOP2A, MARCO, MRC1, MSR1, CD74 | TREM2+dendritic, Platelet/Megakaryocyte, Macrophage, Myeloid dendritic type 2,  IGSF21+ dendritic,  EREG+ dendritic, | TREM2+dendritic, Platelet/Megakaryocyte, Macrophage, IGSF21+ dendritic, | ND | ND | ND | TREM2+dendritic, Platelet/Megakaryocyte, Macrophage, IGSF21+ dendritic | ND | TREM2+dendritic, Platelet/Megakaryocyte,  Nonclassical monocyte, Myeloid dendritic type 2  Myeloid dendritic type 1,Macrophage, Intermediate monocyte,  IGSF21+ dendritic, EREG+ dendritic, |
| TREM2+ Dendritic | GPR183, TREM2, CD14 | Platelet/Megakaryocyte,  Plasmacytoid dendritic,  OLR1+classic monocyte,  Nonclassical monocyte,  Intermediate monocyte,  IGSF21+ dendritic,  EREG+ dendritic,  Classical monocyte | Proliferating macrophage,  Platelet/Megakaryocyte,  Plasmacytoid dendritic,  OLR1+classic monocyte,  Nonclassical monocyte,  Myeloid dendritic type 2,  Myeloid dendritic type 1, Macrophage, Intermediate monocyte,  IGSF21+ dendritic, EREG+ dendritic, Classical monocyte, B cell | Plasmacytoid dendritic,  OLR1+classic monocyte,  Nonclassical monocyte, Myeloid dendritic type 2,  Myeloid dendritic type 1,Macrophage, Intermediate monocyte,  IGSF21+ dendritic, EREG+ dendritic, Classical monocyte, Capillary intermediate endothelia 1, Capillary endothelia,  Bronchial vessel endothelia 2  Bronchial vessel endothelia 1, B cell | OLR1+classic monocyte, Myeloid dendritic type 2,  Myeloid dendritic type 1, Macrophage,Intermediate monocyte,  IGSF21+ dendritic, EREG+ dendritic, Classical monocyte | OLR1+classic monocyte,  Nonclassical monocyte, Myeloid dendritic type 2,  Myeloid dendritic type 1,Macrophage, Intermediate monocyte,  IGSF21+ dendritic, EREG+ dendritic,  Classical monocyte | Plasmacytoid dendritic, OLR1+classic monocyte,  Nonclassical monocyte, Myeloid dendritic type 2,  Myeloid dendritic type 1,Macrophage, Intermediate monocyte,  IGSF21+ dendritic, EREG+ dendritic  , Classical monocyte | Plasmacytoid dendritic, OLR1+classic monocyte, Myeloid dendritic type 2,  Myeloid dendritic type 1,Macrophage, Intermediate monocyte,  IGSF21+ dendritic,  EREG+ dendritic  , Classical monocyte, Capillary intermediate endothelia 1, Capillary endothelia,  Bronchial vessel endothelia 2,  Bronchial vessel endothelia 1, B cell | Plasmacytoid dendritic, OLR1+classic monocyte,  Nonclassical monocyte, Myeloid dendritic type 2,  Myeloid dendritic type 1,Macrophage, Intermediate monocyte, IGSF21+ dendritic, EREG+ dendritic  , Classical monocyte |
