# Supplementary material for: Evaluation of pulmonary single‐cell identity specificity in scRNA‐seq analysis
Source: Clin Transl Med. 2022 Dec 10;12(12):e1132. doi: 10.1002/ctm2.1132 (PMC9736794; doi:10.1002/ctm2.1132)
Supplement: Supplementary file 9 — Supporting Information [file CTM2-12-e1132-s003.docx]

Supplemental Table 7. Overlap expression cell subset of each cell subset marker gene panel of human lung tissues harvested from patients with lung adenocarcinoma (LUAD), large cell cancer (LCC), idiopathic pulmonary fibrosis (IPF), chronic obstructive pulmonary disease (COPD), and systemic sclerosis (SSC) total, normal (Norm), and para-cancer human lung tissues.

| **Cell subset** | **Gene panel** | **Total** | **Normal** | **Para-cancer** | **LCC** | **LUAD** | **IPF** | **COPD** | **SSC** |
| --- | --- | --- | --- | --- | --- | --- | --- | --- | --- |
| Alveolar Epithelial Type 1 | HOPX, PDPN, CLIC5, AGER, CLDN18, EMP2 | 0 | 0 | 0 | Vein endothelia,  Vascular smooth muscle cell, Signaling AT2,  Serous epithelia, Natural killer T cell,  Myofibroblast, Mucous epithelia,  Mesothelial cell,  Lymphatic endothelia, Fibromyocyte,  Club epithelia  , Ciliated epithelia, CD8+ naïve T cell,  CD8+ memory/effector T cell, Capillary intermediate endothelia 2, Capillary aerocyte, Capillary endothelia, Bronchial vessel endothelia 1,  Artery endothelia,  Alveolar fibroblast,  Alveolar epithelial type2, Airway smooth muscle cell,  Adventitial fibroblast | Signaling_AT2, AT2 | AT2 | 0 | 0 |
| Alveolar Epithelial Type 2 | SFTPB, SFTPC, SFTPD, ETV5, MUC1, WIF1, HHIP | Signaling_AT2 | Signaling_AT2 | Signaling_AT2 | Signaling AT2,  Serous epithelia,  Mucous epithelia | Signaling_AT2 | Signaling_AT2 | Signaling_AT2 | Signaling_AT2 |
| Basal | KRT5, KRT14 | Proximal basal cell | Proximal basal cell, Differentiating basal epithelia | 0 | 0 | ND |  | Proximal basal cell, Differentiating basal epithelia | Proximal basal cell, Differentiating basal epithelia |
| Proximal Ciliated | FOXJ1 | Proximal ciliated epithelia, Platelet/Megakaryocyte | Proximal ciliated epithelia | 0 | 0 | 0 | Proximal ciliated epithelia, Platelet/Megakaryocyte | Proximal ciliated epithelia, Platelet/Megakaryocyte | roximal ciliated epithelia, Ionocyte |
| Club | SCGB1A1, SCGB3A2 | Signlaing_AT2, Serous epithelia, Mucous epithelia, Goblet epithelia  , Ciliated epithelia,AT2 | Serous epithelia, Mucous epithelia, Goblet epithelia | Mucous epithelia | Vein endothelia,  Vascular smooth muscle cell, TREM2+dendritic,Signaling AT2,  Serous epithelia,  Proximal basal epithelia, Platelet/Megakaryocyte,  Plasmacytoid dendritic  , Pericyte cell,  OLR1+classic monocyte,  Nonclassical monocyte,  Natural killer T cell,  Natural killer,  Myofibroblast,  Myeloid dendritic type 2,  Myeloid dendritic type 1, Mucous epithelia,  Mesothelial cell,  Macrophage, Lymphatic endothelia, Ionocyte, Goblet epithelia, EREG+ dendritic,  Differentiating basal epithelia, Classical monocyte,  Ciliated epithelia,  CD8+ naïve T cell,  CD8+ memory/effector T cell, CD4+ memory/effector T cell, Basophil/Mast 2,  Basal epithelia,  B cell,  Artery endothelia,  Alveolar fibroblast,  Alveolar epithelial type2,  AT1,  Airway smooth muscle cell,  Adventitial fibroblast | Signaling AT2,  Serous epithelia, Mucous epithelia  , Differentiating basal epithelia  , Ciliated epithelia  ,AT2,AT1 | Signaling AT2,  Serous epithelia, Mucous epithelia, Goblet epithelia | Serous epithelia, Mucous epithelia, Goblet epithelia | Serous epithelia, Mucous epithelia, Goblet epithelia, Differentiating basal epithelia |
| Proximal Basal | KRT7, KRT13 | Serous epithelia, Proximal basal epithelia, Proliferating basal epithelia, Mucous epithelia, Goblet epithelia, Club epithelia, Basal epithelia, AT1 | Signaling_AT2,Serous epithelia, Proximal basal epithelia, Mucous epithelia, Mesothelial cell, Goblet epithelia, Club epithelia, Ciliated epithelia, Basal epithelia,AT2,AT1 | ND | Signaling_AT2, Proximal basal epithelia, Proliferating basal epithelia, Plasma cell  , Mucous epithelia, Goblet epithelia, Club epithelia,Basal epithelia, AT2,AT1 | Proximal basal epithelia, Proliferating basal epithelia, Platelet/Megakaryocyte  , Mucous epithelia, Goblet epithelia, Club epithelia, Basal epithelia,AT1 | Signaling_AT2,Serous epithelia, Proximal basal epithelia, Proliferating basal epithelia, Mucous epithelia, Mesothelial cell, Goblet epithelia, Club epithelia, Ciliated epithelia, Basal epithelia, AT2,AT1 | Signaling_AT2, Serous epithelia, Proximal ciliated epithelia, Proximal basal epithelia, Mucous epithelia, Mesothelial cell, Goblet epithelia,  Fibromyocyte, Club epithelia, Ciliated epithelia, Basal epithelia, AT2,AT1 | Signaling_AT2, Serous epithelia, Proximal ciliated epithelia, Proximal basal epithelia, Platelet/Megakaryocyte, Mucous epithelia, Goblet epithelia, Club epithelia, Ciliated epithelia, Basal epithelia, AT2,AT1 |
| Goblet | MUC5AC, MUC5B | Serous epithelia, Mucous epithelia, | 0 | Mucous epithelia, | Serous epithelia, Platelet/Megakaryocyte,  Mucous epithelia, | Mucous epithelia, Club epithelia | Serous epithelia, Mucous epithelia, | Mucous epithelia, | Serous epithelia, Mucous epithelia |
| Ionocyte | FOXI1, CFTR | ND | ND | ND | ND | ND | ND | ND | ND |
| Mucous | MUC5B, MUC5AC | Serous epithelia, Goblet epithelia | Serous epithelia, Goblet epithelia | Serous epithelia, Goblet epithelia | Serous epithelia, Platelet/Megakaryocyte, Goblet epithelia | Goblet epithelia, Differentiating basal epithelia,  Club epithelia | Serous epithelia, Goblet epithelia | Goblet epithelia | Serous epithelia, Goblet epithelia |
| Neuroendocrine | ASCL1, CHGA | 0 | 0 | ND | ND | ND | ND | 0 | ND |
| Proliferating Basal | MKI67 | ND | ND | ND | ND | ND | ND | ND | ND |
| Proximal Basal | KRT5, KRT14 | Basal epithelia | Differentiating basal epithelia, Basal epithelia | ND | ND | ND | Differentiating basal epithelia, Basal epithelia | Differentiating basal epithelia, Basal epithelia | Differentiating basal epithelia, Basal epithelia |
| Proximal Ciliated | FOXJ1 | Ciliated epithelia | Ciliated epithelia | ND | ND | ND | Ciliated epithelia | Ciliated epithelia | Ciliated epithelia |
| Serous | LPO, LTF | ND | ND | 0 | Singaling_AT2, Mucous epithelia | Proximal basal epithelia, Proliferating basal epithelia,  Platelet/Megakaryocyte, Ionocyte | ND | Proximal basal epithelia, Mucous epithelia, Goblet epithelia,Fibromyocyte | ND |
| Signaling Alveolar Epithelial Type 2 | CP, SFTPC | AT2 | AT2 | AT2 | 0 | Vein endothelia, Serous epithelia, Platelet/Megakaryocyte,  Plasmacytoid dendritic, OLR1+classic monocyte, Myeloid dendritic type 1,  Mucous epithelia, Macrophage,  Lymphatic endothelia,  Lipofibroblast,  Ionocyte, IGSF21+ dendritic,  Goblet epithelia,  Fibromyocyte, Differentiating basal epithelia,  Club epithelia, Ciliated epithelia, CD4+ memory/effector T cell,  Capillary intermediate endothelia 2,  Capillary intermediate endothelia 1,  Capillary aerocyte, Bronchial vessel endothelia 1,  Basophil/Mast 2,  Basophil/Mast 1, B cell,  Artery endothelia,  Alveolar fibroblast,  Alveolar epithelial type2 | Serous epithelia, Mucous epithelia, Goblet epithelia,  AT2 | AT2 | AT2 |
