# Supplementary material for: Evaluation of pulmonary single‐cell identity specificity in scRNA‐seq analysis
Source: Clin Transl Med. 2022 Dec 10;12(12):e1132. doi: 10.1002/ctm2.1132 (PMC9736794; doi:10.1002/ctm2.1132)
Supplement: Supplementary file 10 — Supporting Information [file CTM2-12-e1132-s006.docx]

Supplemental Table 8. Overlap expression cell subset of each cell subset marker gene panel of human lung tissues harvested from patients with lung adenocarcinoma (LUAD), large cell cancer (LCC), idiopathic pulmonary fibrosis (IPF), chronic obstructive pulmonary disease (COPD), and systemic sclerosis (SSC) total, normal (Norm), and para-cancer human lung tissues.

| **Cell subset** | **Gene panel** | **Total** | **Normal** | **Para-cancer** | **LCC** | **LUAD** | **IPF** | **COPD** | **SSC** |
| --- | --- | --- | --- | --- | --- | --- | --- | --- | --- |
| Artery | CXCL12, DKK2, GJA5, SERPINE2 | 0 | 0 | 0 | Myofibroblast, Adventitial fibroblast | 0 | 0 | 0 | Lipofibroblast |
| Bronchial Vessel 1 | MYC, SPRY1 | Vein endothelia  Vascular smooth muscle cell, Proximal basal epithelia, Pericyte cell, Neuroendocrine epithelia, Myofibroblast, Mesothelial cell, Lymphatic endothelia  Lipofibroblast, Fibromyocyte, Differentiating basal epithelia, Capillary intermediate endothelia 2,Capillary endothelia  , Basal epithelia, Artery endothelia  Alveolar fibroblast  Alveolar epithelial type2, Airway smooth muscle cell  Adventitial fibroblast | Vein endothelia  Vascular smooth muscle cell, Proximal basal epithelia, Pericyte cell, Neuroendocrine epithelia, Myofibroblast, Mesothelial cell, Lymphatic endothelia  Lipofibroblast, Fibromyocyte, Differentiating basal epithelia, Capillary intermediate endothelia 2,Capillary endothelia  , Basal epithelia, Artery endothelia  Alveolar fibroblast  Alveolar epithelial type2, Airway smooth muscle cell  Adventitial fibroblast | ____ | Vein endothelia, Lymphatic endothelia | Vein endothelia  Vascular smooth muscle cell, Proximal basal epithelia, Pericyte cell, Neuroendocrine epithelia, Myofibroblast, Mesothelial cell, Lymphatic endothelia  Lipofibroblast, Fibromyocyte, Differentiating basal epithelia, Capillary intermediate endothelia 2,Capillary endothelia  , Basal epithelia, Artery endothelia  Alveolar fibroblast  Alveolar epithelial type2, Airway smooth muscle cell  Adventitial fibroblast | Vein endothelia, Capillary intermediate endothelia 2,  Capillary endothelia | Vein endothelia  Vascular smooth muscle cell, Proximal basal epithelia, Pericyte cell, Neuroendocrine epithelia, Myofibroblast, Mesothelial cell, Lymphatic endothelia  Lipofibroblast, Fibromyocyte, Differentiating basal epithelia, Capillary intermediate endothelia 2,Capillary endothelia  , Basal epithelia, Artery endothelia  Alveolar fibroblast  Alveolar epithelial type2, Airway smooth muscle cell  Adventitial fibroblast | Vein endothelia  Vascular smooth muscle cell, Proximal basal epithelia, Pericyte cell, Neuroendocrine epithelia, Myofibroblast, Mesothelial cell, Lymphatic endothelia  Lipofibroblast, Fibromyocyte, Differentiating basal epithelia, Capillary intermediate endothelia 2,Capillary endothelia  , Basal epithelia, Artery endothelia  Alveolar fibroblast  Alveolar epithelial type2, Airway smooth muscle cell  Adventitial fibroblast |
| Bronchial Vessel 2 | MYC | ND | ND | ND | ND | ND | ND | ND | ND |
| Capillary | IL7R, SLC6A4, FCN3 | CD4+ naïve T cell,  CD4+ memory/effector T cell,  Capillary intermediate endothelia 2,  Capillary intermediate endothelia 1,  Bronchial vessel endothelia 2 | Capillary intermediate endothelia 2,  Capillary intermediate endothelia 1,  Bronchial vessel endothelia 2,  Bronchial vessel endothelia 1 | Capillary intermediate endothelia 1,  Bronchial vessel endothelia 2,  Bronchial vessel endothelia 1 | Proliferating NK/T cell,  Natural killer T cell,  Natural killer,  CD8+ naïve T cell,  CD8+ memory/effector T cell,  CD4+ naïve T cell,  CD4+ memory/effector T cell,  Capillary intermediate endothelia 2 | Natural killer T cell, CD8+ memory/effector T cell,  CD4+ naïve T cell,  CD4+ memory/effector T cell,  Capillary intermediate endothelia 2,  Capillary intermediate endothelia 1,  Capillary aerocyte, Bronchial vessel endothelia 1 | Proliferating NK/T cell,  Proliferating macrophage,  Natural killer T cell,  Natural killer,  CD8+ naïve T cell,  CD8+ memory/effector T cell,  CD4+ naïve T cell,  CD4+ memory/effector T cell,  Capillary intermediate endothelia 2,  Capillary intermediate endothelia 1,  Capillary aerocyte,  Bronchial vessel endothelia 2,  Artery endothelia | Capillary intermediate endothelia 2,  Capillary intermediate endothelia 1,  Bronchial vessel endothelia 2,  Bronchial vessel endothelia 1 | Capillary intermediate endothelia 1, Bronchial vessel endothelia 2 |
| Capillary Aerocyte | APLN, EDNRB, HPGD | 0 | Capillary intermediate endothelia 1 | Capillary intermediate endothelia 1 | 0 | 0 | 0 | Capillary intermediate endothelia 1 | Capillary intermediate endothelia 1 |
| Capillary Intermediate 1 | APLN, EDNRB, HPGD, IL1RL1 | Capillary intermediate endothelia 2,  Capillary aerocyte ,Bronchial vessel endothelia 1,  Basophil/Mast 2,  Basophil/Mast 1 | Capillary intermediate endothelia 2,  Capillary aerocyte, Basophil/Mast 2 | Capillary intermediate endothelia 2,  Capillary aerocyte | ND | Capillary aerocyte, Basophil/Mast 2,  Basophil/Mast 1 | TREM2+dendritic, Basophil/Mast 2,  Basophil/Mast 1 | Capillary aerocyte, Basophil/Mast 2,  Basophil/Mast 1 | Pericyte cell, Capillary intermediate endothelia 2,  Capillary aerocyte,  Capillary endothelia,  Bronchial vessel endothelia 2,  Basophil/Mast 2,  Basophil/Mast 1 |
| Capillary Intermediate 2 | IL7R, SLC6A4, FCN3 | Vein endothelia,  Proliferating NK/T cell,Proliferating macrophage, OLR1+classic monocyte,  Natural killer T cell,  Myeloid dendritic type 1,  Macrophage,  CD8+ naïve T cell,  CD8+ memory/effector T cell,  CD4+ naïve T cell,  CD4+ memory/effector T cell,  Capillary intermediate endothelia 1,  Capillary aerocyte,  Capillary endothelia,  Bronchial vessel endothelia 2,  Bronchial vessel endothelia 1,  Artery endothelia | Vein endothelia,  Proliferating NK/T cell, Proliferating macrophage, Natural killer T cell, CD8+ memory/effector T cell,  CD4+ naïve T cell,Capillary intermediate endothelia 1,  Capillary aerocyte,  Capillary endothelia,  Bronchial vessel endothelia 2,  Bronchial vessel endothelia 1,  Artery endothelia | Vein endothelia,  Proliferating NK/T cell, Plasma cell, Natural killer T cell,  Natural killer,CD8+ naïve T cell,  CD8+ memory/effector T cell,  CD4+ naïve T cell,  CD4+ memory/effector T cell,  Capillary intermediate endothelia 1,  Capillary aerocyte,  Capillary endothelia,  Bronchial vessel endothelia 2,  Bronchial vessel endothelia 1,  B cell,  Artery endothelia | Proliferating NK/T cell, Natural killer T cell,  Natural killer, Myeloid dendritic type 2,  Myeloid dendritic type 1, EREG+ dendritic, CD8+ naïve T cell,  CD8+ memory/effector T cell,  CD4+ naïve T cell,  CD4+ memory/effector T cell, Capillary endothelia, Basophil/Mast 2 | Vein endothelia,  TREM2+ dendritic, Natural killer T cell, Myeyloid dendritic type 2, Myeloid dendritic type 1,Macrophage, EREG+dendritic, CD8+ memory/effector T cell,  CD4+ naïve T cell,  CD4+ memory/effector T cell, Capillary intermediate endothelia 1,  Capillary aerocyte,  Capillary endothelia  , Bronchial vessel endothelia 1, Artery endothelia | Vein endothelia,  TREM2+ dendritic, Proliferating NK/T cell, Proliferating macrophage, Platelet/Megakaryocyte, OLR1+classical monocyte, Natural killer T cell,  Natural killer, Myeyloid dendritic type 2, Myeloid dendritic type 1, Macrophage,  EREG+dendritic, CD8+ naïve T cell,  CD8+ memory/effector T cell,  CD4+ naïve T cell,  CD4+ memory/effector T cell, Capillary intermediate endothelia 1,  Capillary aerocyte,  Capillary endothelia,  Bronchial vessel endothelia 2,  Artery endothelia | Proliferating NK/T cell, Platelet/Megakaryoc, Natural killer T cell,  Natural killer, Macrophage, CD8+ memory/effectorT cell,  CD4+ naïve T cell,  CD4+ memory/effector T cell, Capillary intermediate endothelia 1,  Capillary aerocyte,  Capillary endothelia,  Bronchial vessel endothelia 2,  Bronchial vessel endothelia 1 | Vein endothelia,  Proliferating NK/T cell, OLR1+classic monocyte, Natural killer T cell,  Natural killer, Myeloid dendritic type 1, Macrophage, CD8+ naïve T cell,  CD8+ memory/effector T cell,  CD4+ naïve T cell,  CD4+ memory/effector T cell, Capillary intermediate endothelia 1,  Capillary aerocyte,  Capillary endothelia,  Bronchial vessel endothelia 2,  Bronchial vessel endothelia 1,  Artery endothelia |
| Lymphatic | TFF3, PDPN, CCL21, IGF1 | 0 | 0 | 0 | Mesothelial cell, Ionocyte, Goblet epithelia,  Fibromyocyte, Alveolar fibroblast, Airway smooth muscle cell,  Adventitial fibroblast | 0 | 0 | 0 | 0 |
| Vein | PTGIS, CPE | Vascular smooth muscle cell, Pericyte cell, Myofibroblast, Mesothelial cell, Lipofibroblast, Fibromyocyte, Alveolar fibroblast, Airway smooth muscle cell,  Adventitial fibroblast | Neuroendocrine epithelia, Mesothelial cell, Artery endothelia, Airway smooth muscle cell,  Adventitial fibroblast | Adventitial fibroblast | Vascular smooth muscle cell, Proximal basal epithelia, Proliferating basal epithelia, Platelet/Megakaryocyte, Pericyte cell, Myofibroblast, Mucous epithelia  Mesothelial cell, Ionocyte,  Goblet epithelia, Differentiating basal epithelia  Club epithelia, Basal epithelia, Alveolar fibroblast  Alveolar epithelial type2  Alveolar epithelial type1  Airway smooth muscle cell  Adventitial fibroblast | Vascular smooth muscle cell, Pericyte cell, Myofibroblast, Mesothelial cell, Lipofibroblast, Fibromyocyte, Alveolar fibroblast  Airway smooth muscle cell,  Adventitial fibroblast | Vascular smooth muscle cell, Pericyte cell, Myofibroblast, Mesothelial cell, Fibromyocyte, Bronchial vessel endothelia 2, Artery endothelia, Alveolar fibroblast, Airway smooth muscle cell,  Adventitial fibroblast | Vascular smooth muscle cell, Pericyte cell, Neuroendocrine epithelia, Myofibroblast, Mesothelial cell, Fibromyocyte, Artery endothelia, Alveolar fibroblast, Airway smooth muscle cell,  Adventitial fibroblast | Vascular smooth muscle cell, Myofibroblast, Lipofibroblast, Fibromyocyte, Artery endothelia, Alveolar fibroblast, Airway smooth muscle cell,  Adventitial fibroblast |
