# Supplementary material for: Evaluation of pulmonary single‐cell identity specificity in scRNA‐seq analysis
Source: Clin Transl Med. 2022 Dec 10;12(12):e1132. doi: 10.1002/ctm2.1132 (PMC9736794; doi:10.1002/ctm2.1132)
Supplement: Supplementary file 11 — Supporting Information [file CTM2-12-e1132-s007.docx]

Supplemental Table 9. Overlap expression cell subset of each cell subset marker gene panel of human lung tissues harvested from patients with lung adenocarcinoma (LUAD), large cell cancer (LCC), idiopathic pulmonary fibrosis (IPF), chronic obstructive pulmonary disease (COPD), and systemic sclerosis (SSC) total, normal (Norm), and para-cancer human lung tissues.

| **Cell subset** | **Gene panel** | **Total** | **Normal** | **Para-cancer** | **LCC** | **LUAD** | **IPF** | **COPD** | **SSC** |
| --- | --- | --- | --- | --- | --- | --- | --- | --- | --- |
| Adventitial Fibroblast | SERPINF1, PI16, FBLN1, SCARA5 | 0 | 0 | 0 | Myofibroblast,  Alveolar fibroblast | Lipofibroblast,  Adventitial fibroblast | Alveolar fibroblast | 0 | Alveolar fibroblast |
| Airway Smooth Muscle | ACTA2, TAGLN, CNN1, MYH11, DES, KCNA5 | Vascular smooth muscle cell | Vascular smooth muscle cell, Fibromyocyte | 0 | Vascular smooth muscle cell | 0 | Vascular smooth muscle cell | Vascular smooth muscle cell | Vascular smooth muscle cell |
| Alveolar Fibroblast | SLC38A5, GDF10, GPC3, ITGA8 | Lipofibroblast | Fibromyocyte, Adventitial fibroblast | 0 | Proliferating basal epithelia,  Plasmacytoid dendritic, Myofibroblast,  Fibromyocyte,  Ciliated epithelia, Capillary aerocyte, Basal epithelia, AT2, AT1, Adventitial fibroblast | Lipofibroblast | Lipofibroblast | 0 | Lipofibroblast |
| Fibromyocyte | ASPN, FGF18, ACTA2, TAGLN, CNN1 | Vascular smooth muscle cell, Myofibroblast, Airway smooth muscle cell | Vascular smooth muscle cell, Pericyte cell, Myofibroblast, Airway smooth muscle cell | ND | Vascular smooth muscle cell, Myofibroblast, Airway smooth muscle cell | Vascular smooth muscle cell, Myofibroblast, Airway smooth muscle cell | Vascular smooth muscle cell, Myofibroblast, Airway smooth muscle cell | Vascular smooth muscle cell, Proximal basal epithelia, Pericyte cell, Myofibroblast,  Artery endothelia, Airway smooth muscle cell, Adventitial fibroblast | 7.14 ascular smooth muscle cell, Pericyte cell, Myofibroblast, Airway smooth muscle cell |
| Lipofibroblast | PLIN2, APOE | Vein endothelia  Vascular smooth muscle cell  TREM2+dendritic  Signaling AT2  Proliferating macrophage  Platelet/Megakaryocyte  Plasmacytoid dendritic  Plasma cell  Pericyte cell  OLR1+classic monocyte  Nonclassical monocyte  Neuroendocrine epithelia  Natural killer T cell  Natural killer  Myofibroblast  Myeloid dendritic type 2  Myeloid dendritic type 1  Mesothelial cell  Macrophage  Lymphatic endothelia  Lipofibroblast  Ionocyte  Intermediate monocyte  IGSF21+ dendritic  Fibromyocyte  EREG+ dendritic  Classical monocyte  CD8+ naïve T cell  CD8+ memory/effector T cell  CD4+ memory/effector T cell  Capillary intermediate endothelia 2  Capillary intermediate endothelia 1  Capillary aerocyte  Capillary endothelia  Bronchial vessel endothelia 2  Bronchial vessel endothelia 1  Basophil/Mast 2  Basophil/Mast 1  B cell  Artery endothelia  Alveolar fibroblast  Alveolar epithelial type2  Alveolar epithelial type1  Airway smooth muscle cell  Adventitial fibroblast | ND | ND | ND | Vein endothelia  Vascular smooth muscle cell  TREM2+dendritic  Signaling AT2  Serous epithelia  Proximal ciliated epithelia  Proximal basal epithelia  Proliferating basal epithelia  Platelet/Megakaryocyte  Plasmacytoid dendritic  Plasma cell  Pericyte cell  OLR1+classic monocyte  Nonclassical monocyte  Neuroendocrine epithelia  Natural killer T cell  Natural killer  Myofibroblast  Myeloid dendritic type 2  Myeloid dendritic type 1  Mucous epithelia  Mesothelial cell  Macrophage  Lymphatic endothelia  Ionocyte  Intermediate monocyte  IGSF21+ dendritic  Goblet epithelia  Fibromyocyte  EREG+ dendritic  Differentiating basal epithelia  Club epithelia  Classical monocyte  CD8+ naïve T cell  CD8+ memory/effector T cell  CD4+ memory/effector T cell  Capillary intermediate endothelia 2  Capillary intermediate endothelia 1  Capillary aerocyte  Capillary endothelia  Bronchial vessel endothelia 1  Basophil/Mast 2  Basophil/Mast 1  Basal epithelia  B cell  Artery endothelia  Alveolar fibroblast  Alveolar epithelial type2  Alveolar epithelial type1  Airway smooth muscle cell  Adventitial fibroblast | Vein endothelia  Vascular smooth muscle cell  TREM2+dendritic  Signaling AT2  Proximal ciliated epithelia  Proximal basal epithelia  Proliferating NK/T cell  Proliferating macrophage  Platelet/Megakaryocyte  Plasmacytoid dendritic  Plasma cell  Pericyte cell  OLR1+classic monocyte  Nonclassical monocyte  Neuroendocrine epithelia  Natural killer T cell  Natural killer  Myofibroblast  Myeloid dendritic type 2  Myeloid dendritic type 1  Mucous epithelia  Mesothelial cell  Macrophage  Lymphatic endothelia  Ionocyte  Intermediate monocyte  IGSF21+ dendritic  Goblet epithelia  Fibromyocyte  EREG+ dendritic  Differentiating basal epithelia  Club epithelia  Classical monocyte  Ciliated epithelia  CD8+ naïve T cell  CD8+ memory/effector T cell  CD4+ naïve T cell  CD4+ memory/effector T cell  Capillary intermediate endothelia 2  Capillary intermediate endothelia 1  Capillary aerocyte  Capillary endothelia  Bronchial vessel endothelia 2  Bronchial vessel endothelia 1  Basophil/Mast 2  Basophil/Mast 1  Basal epithelia  B cell  Artery endothelia  Alveolar fibroblast  Alveolar epithelial type2  Alveolar epithelial type1  Airway smooth muscle cell  Adventitial fibroblast | ND | Vascular smooth muscle cell, Proliferating macrophage, Platelet/Megakaryocyte, Plasma cell  Pericyte cell,  OLR1+classic monocyte,  Nonclassical monocyte, Myofibroblast,  Myeloid dendritic type 2,  Myeloid dendritic type 1, Macrophage,  Intermediate monocyte,  IGSF21+ dendritic, Fibromyocyte,  EREG+ dendritic, Classical monocyte, CD4+ memory/effector T cell, Basophil/Mast 2,  Basophil/Mast 1,B cell, Alveolar fibroblast, Adventitial fibroblast |
| Mesothelial | MSLN, KRT19, UPK3B | AT1 | 0 | AT1 | Signaling_AT2, Proximal basal epithelia, Proliferating basal epithelia,  Platelet/Megakaryocyte, Mucous epithelia, Ionocyte, Goblet epithelia, Differentiating basal epithelia,  Club epithelia, Basal epithelia, AT1,AT2 | Proximal basal epithelia, Goblet epithelia, Differentiating basal epithelia,  Club epithelia, Basal epithelia, AT1 | 0 | 0 | ND |
| Myofibroblast | ACTA2, MYH11, ASPN, TYRP1 | Vascular smooth muscle cell, Pericyte cell, Fibromyocyte, Airway smooth muscle cell | Vascular smooth muscle cell, Pericyte cell, Fibromyocyte, Alveolar fibroblast,Airway smooth muscle cell | Vascular smooth muscle cell | Vascular smooth muscle cell, Pericyte cell, Fibromyocyte, Airway smooth muscle cell | Vascular smooth muscle cell, Pericyte cell, Lipofibroblast, Fibromyocyte, Airway smooth muscle cell | Vascular smooth muscle cell, Pericyte cell, Fibromyocyte, Alveolar fibroblast,Airway smooth muscle cell, Adventitial fibroblast | Vascular smooth muscle cell, Proximal ciliated epithelia, Pericyte cell, Fibromyocyte, Airway smooth muscle cell, Adventitial fibroblast | Vascular smooth muscle cell, Pericyte cell, Fibromyocyte, Airway smooth muscle cell |
| Pericyte | COX4I2, HIGD1B, GJA4 | 0 | 0 | 0 | Airway smooth muscle cell | 0 | 0 | 0 | Vascular smooth muscle cell |
| Vascular Smooth Muscle | ACTA2, TAGLN, CNN1, MYH11 | Fibromyocyte,  Airway smooth muscle cell | Pericyte cell,Fibromyocyte, Airway smooth muscle cell | Pericyte cell, Airway smooth muscle cell | Airway smooth muscle cell | Fibromyocyte, Airway smooth muscle cell | Fibromyocyte, Airway smooth muscle cell | Airway smooth muscle cell | Fibromyocyte, Airway smooth muscle cell |
