# Supplementary material for: Evaluation of pulmonary single‐cell identity specificity in scRNA‐seq analysis
Source: Clin Transl Med. 2022 Dec 10;12(12):e1132. doi: 10.1002/ctm2.1132 (PMC9736794; doi:10.1002/ctm2.1132)
Supplement: Supplementary file 12 — Supporting Information [file CTM2-12-e1132-s010.docx]

Overlap expression cell subset of each cell subset marker gene panel of human lung tissues harvested from patients with lung adenocarcinoma (LUAD), large cell cancer (LCC), idiopathic pulmonary fibrosis (IPF), chronic obstructive pulmonary disease (COPD), and systemic sclerosis (SSC) total, normal (Norm), and para-cancer human lung tissues.

| **Cell subset** | **Gene panel** | **Total** | **Normal** | **Para-cancer** | **LCC** | **LUAD** | **IPF** | **COPD** | **SSC** |
| --- | --- | --- | --- | --- | --- | --- | --- | --- | --- |
| CD4+ Memory/Effector T | CD3E, CD4, LTB, COTL1, LDHB | Proliferating NK/T cell, Proliferating macrophage,  Platelet/Megakaryocyte, Nonclassical monocyte, Natural killer T cell,  Natural killer, Myeyloid dendritic type 2, Myeloid dendritic type 1, Macrophage, Intermediate monocyte, EREG+ dendritic, classic monocyte, CD8+ naïve T cell,  CD8+ memory/effector T cell,  CD4+ naïve T cell,  B cell | TREM2+dendrtitic, Proximal ciliated epithelia  , Proliferating macrophage,  Proliferating basal epithelia,  Platelet/Megakaryocyte,  OLR1+classic monocyte,  Nonclassical monocyte,  Natural killer T cell,  Natural killer,  Myeloid dendritic type 2,  Myeloid dendritic type 1, Macrophage, Intermediate monocyte,  IGSF21+ dendritic, EREG+dendritic, Classical monocyte,  CD8+ naïve T cell,  CD8+ memory/effector T cell,  CD4+ naïve T cell, B cell | Proliferating NK/T cell, Nonclassical monocyte,  Natural killer T cell,  Natural killer,  Myeloid dendritic type 2,  Myeloid dendritic type 1,  Intermediate monocyte,  EREG+ dendritic,  CD8+ naïve T cell,  CD8+ memory/effector T cell,  CD4+ naïve T cell,  Basophil/Mast 2 | Proliferating NK/T cell, Nonclassical monocyte  , Natural killer T cell,  Natural killer  ,Myeloid dendritic type 2,  Myeloid dendritic type 1,  Intermediate monocyte,  EREG+ dendritic,  CD8+ naïve T cell,  CD8+ memory/effector T cell,  CD4+ naïve T cell, B cell | Natural killer T cell,  Natural killer, Myeloid dendritic type 2,  Myeloid dendritic type 1, CD8+ naïve T cell,  CD8+ memory/effector T cell,  CD4+ naïve T cell, | Vein endothelia, TREM2+dendrtitic, Proliferating NK/T cell, Proliferating macrophage,  Platelet/Megakaryocyte,  Plasmacytoid dendritic,  OLR1+classic monocyte,  Nonclassical monocyte,  Natural killer T cell, Myeloid dendritic type 2,  Myeloid dendritic type 1, Macrophage, Lipofibroblast, Intermediate monocyte,  IGSF21+ dendritic, EREG+ dendritic, Classical monocyte, CD8+ naïve T cell,  CD8+ memory/effector T cell,  CD4+ naïve T cell,  Basophil/Mast 2,  Basophil/Mast 1,B cell | Vein endothelia,  Vascular smooth muscle cell,  Signaling AT2,  Serous epithelia,  Proximal ciliated epithelia,  Proximal basal epithelia,  Proliferating NK/T cell,  Proliferating macrophage,  Proliferating basal epithelia,  Platelet/Megakaryocyte,  Plasmacytoid dendritic,  Plasma cell,  Pericyte cell,  OLR1+classic monocyte,  Nonclassical monocyte,  Neuroendocrine epithelia,  Natural killer T cell,  Natural killer,  Myofibroblast,  Myeloid dendritic type 2,  Myeloid dendritic type 1,  Mucous epithelia,  Mesothelial cell,  Macrophage,  Lipofibroblast,  Ionocyte,  Intermediate monocyte,  IGSF21+ dendritic,  Goblet epithelia,  EREG+ dendritic,  Differentiating basal epithelia,  Club epithelia,  Classical monocyte,  Ciliated epithelia,  CD8+ naïve T cell,  CD8+ memory/effector T cell,  CD4+ naïve T cell,  Capillary intermediate endothelia 1,  Capillary endothelia,  Bronchial vessel endothelia 1,  Basophil/Mast 2,  Basophil/Mast 1,  Basal epithelia,  B cell,  Alveolar fibroblast,  Alveolar epithelial type2,  Airway smooth muscle cell,  Adventitial fibroblast | 25 Platelet/Megakaryocyte,  Plasmacytoid dendritic, Natural killer T cell, Myeloid dendritic type 2,  Myeloid dendritic type 1,Macrophage,  Lipofibroblast,  Intermediate monocyte,  Fibromyocyte,  EREG+ dendritic  , CD8+ naïve T cell,  CD8+ memory/effector T cell, B cell |
| CD4+ Naive T | CD3E, CD4, LTB, LDHB, CCR7, LEF1 | Vein endothelia  Vascular smooth muscle cell  TREM2+dendritic  Signaling AT2  Proximal ciliated epithelia  Proximal basal epithelia  Proliferating NK/T cell  Proliferating macrophage  Proliferating basal epithelia  Platelet/Megakaryocyte  Plasmacytoid dendritic  Pericyte cell  Nonclassical monocyte  Neuroendocrine epithelia  Natural killer T cell  Natural killer  Myofibroblast  Myeloid dendritic type 2  Myeloid dendritic type 1  Mesothelial cell  Macrophage  Lipofibroblast  Ionocyte  Intermediate monocyte  IGSF21+ dendritic  Goblet epithelia  Fibromyocyte  EREG+ dendritic  Differentiating basal epithelia  Club epithelia  Ciliated epithelia  CD8+ naïve T cell  CD8+ memory/effector T cell  CD4+ memory/effector T cell  Bronchial vessel endothelia 1  Basophil/Mast 2  Basophil/Mast 1  Basal epithelia  B cell  Alveolar fibroblast  Alveolar epithelial type2  Airway smooth muscle cell  Adventitial fibroblast | Vein endothelia  Vascular smooth muscle cell  TREM2+dendritic  Signaling AT2  Proximal ciliated epithelia  Proximal basal epithelia  Proliferating NK/T cell  Proliferating macrophage  Proliferating basal epithelia  Platelet/Megakaryocyte  Plasmacytoid dendritic  Pericyte cell  OLR1+classic monocyte  Nonclassical monocyte  Natural killer T cell  Natural killer  Myofibroblast  Myeloid dendritic type 2  Myeloid dendritic type 1  Mucous epithelia  Mesothelial cell  Macrophage  Ionocyte  Intermediate monocyte  IGSF21+ dendritic  Goblet epithelia  Fibromyocyte  EREG+ dendritic  Differentiating basal epithelia  Club epithelia  Classical monocyte  Ciliated epithelia  CD8+ naïve T cell  CD8+ memory/effector T cell  CD4+ memory/effector T cell  Bronchial vessel endothelia 1  Basophil/Mast 2  Basophil/Mast 1  Basal epithelia  B cell  Artery endothelia  Alveolar fibroblast  Alveolar epithelial type2  Airway smooth muscle cell  Adventitial fibroblast | Vascular smooth muscle cell  TREM2+dendritic  Signaling AT2  Serous epithelia  Proximal ciliated epithelia  Proximal basal epithelia  Proliferating NK/T cell  Proliferating macrophage  Proliferating basal epithelia  Plasmacytoid dendritic  Plasma cell  Pericyte cell  OLR1+classic monocyte  Nonclassical monocyte  Neuroendocrine epithelia  Natural killer T cell  Natural killer  Myofibroblast  Myeloid dendritic type 2  Myeloid dendritic type 1  Mesothelial cell  Macrophage  Lipofibroblast  Ionocyte  Intermediate monocyte  IGSF21+ dendritic  Goblet epithelia  Fibromyocyte  EREG+ dendritic  Differentiating basal epithelia  Club epithelia  Classical monocyte  Ciliated epithelia  CD8+ naïve T cell  CD8+ memory/effector T cell  CD4+ memory/effector T cell  Basophil/Mast 2  Basophil/Mast 1  Basal epithelia  B cell  Alveolar fibroblast  Alveolar epithelial type2  Airway smooth muscle cell  Adventitial fibroblast | Proliferating NK/T cell, Natural killer T cell,  Natural killer, Myeloid dendritic type 1, CD8+ naïve T cell,  CD8+ memory/effector T cell, CD4+ memory/effector T cell,B cell | Proliferating NK/T cell, Natural killer T cell,  Natural killer, Myeloid dendritic type 1, CD8+ naïve T cell,  CD8+ memory/effector T cell, CD4+ memory/effector T cell,B cell | Vein endothelia  Vascular smooth muscle cell  TREM2+dendritic  Signaling AT2  Proximal ciliated epithelia  Proximal basal epithelia  Proliferating NK/T cell  Proliferating macrophage  Proliferating basal epithelia  Platelet/Megakaryocyte  Plasmacytoid dendritic  Pericyte cell  Nonclassical monocyte  Neuroendocrine epithelia  Natural killer T cell  Natural killer  Myofibroblast  Myeloid dendritic type 2  Myeloid dendritic type 1  Mesothelial cell  Macrophage  Lipofibroblast  Ionocyte  Intermediate monocyte  IGSF21+ dendritic  Goblet epithelia  Fibromyocyte  EREG+ dendritic  Differentiating basal epithelia  Club epithelia  Ciliated epithelia  CD8+ naïve T cell  CD8+ memory/effector T cell  CD4+ memory/effector T cell  Bronchial vessel endothelia 1  Basophil/Mast 2  Basophil/Mast 1  Basal epithelia  B cell  Alveolar fibroblast  Alveolar epithelial type2  Airway smooth muscle cell  Adventitial fibroblast | Vein endothelia  Vascular smooth muscle cell  TREM2+dendritic  Signaling AT2  Serous epithelia  Proximal ciliated epithelia  Proximal basal epithelia  Proliferating NK/T cell  Proliferating basal epithelia  Platelet/Megakaryocyte  Plasmacytoid dendritic  Plasma cell  Pericyte cell  Natural killer T cell  Natural killer  Myofibroblast  Myeloid dendritic type 2  Myeloid dendritic type 1  Mesothelial cell  Macrophage  Intermediate monocyte  IGSF21+ dendritic  Goblet epithelia  Fibromyocyte  EREG+ dendritic  Differentiating basal epithelia  Club epithelia  Ciliated epithelia  CD8+ naïve T cell  CD8+ memory/effector T cell  CD4+ memory/effector T cell  Bronchial vessel endothelia 1  Basophil/Mast 2  Basophil/Mast 1  Basal epithelia  B cell  Alveolar fibroblast  Alveolar epithelial type2  Airway smooth muscle cell  Adventitial fibroblast | Vein endothelia  Vascular smooth muscle cell  TREM2+dendritic  Signaling AT2  Serous epithelia  Proximal ciliated epithelia  Proximal basal epithelia  Proliferating NK/T cell  Proliferating basal epithelia  Platelet/Megakaryocyte  Plasmacytoid dendritic  Pericyte cell  Neuroendocrine epithelia  Natural killer T cell  Natural killer  Myofibroblast  Myeloid dendritic type 2  Myeloid dendritic type 1  Mesothelial cell  Macrophage  Lipofibroblast  IGSF21+ dendritic  Fibromyocyte  EREG+ dendritic  Differentiating basal epithelia  Club epithelia  Ciliated epithelia  CD8+ naïve T cell  CD8+ memory/effector T cell  CD4+ memory/effector T cell  Bronchial vessel endothelia 1  Basophil/Mast 2  Basophil/Mast 1  Basal epithelia  B cell  Alveolar fibroblast  Alveolar epithelial type2  Airway smooth muscle cell  Adventitial fibroblast |
| CD8+ Memory/Effector T | CD3E, GZMK, KLRB1, IL7R, DUSP2 | Proliferating NK/T cell, Natural killer T cell,  Natural killer, CD8+ naïve T cell, CD4+ naïve T cell,  CD4+ memory/effector T cell | Proliferating NK/T cell, Natural killer T cell,  Natural killer, CD8+ naïve T cell, CD4+ naïve T cell,  CD4+ memory/effector T cell | Proliferating NK/T cell, Natural killer T cell,  Natural killer, CD8+ naïve T cell, CD4+ naïve T cell,  CD4+ memory/effector T cell | Proliferating NK/T cell, Natural killer T cell,  Natural killer, CD8+ naïve T cell, CD4+ naïve T cell,  CD4+ memory/effector T cell | Proliferating NK/T cell, Natural killer T cell,  Natural killer, CD8+ naïve T cell, CD4+ naïve T cell,  CD4+ memory/effector T cell | Proliferating NK/T cell, Natural killer T cell,  Natural killer, CD8+ naïve T cell, CD4+ naïve T cell,  CD4+ memory/effector T cell | Proliferating NK/T cell, Natural killer T cell,  Natural killer, CD8+ naïve T cell, CD4+ naïve T cell,  CD4+ memory/effector T cell | Natural killer T cell,  Natural killer, CD8+ naïve T cell, CD4+ naïve T cell,  CD4+ memory/effector T cell |
| CD8+ Naive T | CD3E, GZMH, GZMB | Natural killer T cell,  Natural killer,  CD8+ memory/effector T cell | Natural killer T cell,  Natural killer,  CD8+ memory/effector T cell | Proliferating NK/T cell, Natural killer T cell,  Natural killer, CD8+ memory/effector T cell, CD4+ memory/effector T cell | Proliferating NK/T cell, Natural killer T cell,  Natural killer, CD8+ memory/effector T cell, CD4+ memory/effector T cell | Proliferating NK/T cell, Natural killer T cell,  Natural killer, CD8+ memory/effector T cell, CD4+ memory/effector T cell | Natural killer T cell,  Natural killer, CD8+ memory/effector T cell, CD4+ memory/effector T cell | Natural killer T cell,  Natural killer, CD8+ memory/effector T cell | Proliferating NK/T cell, Natural killer T cell,  Natural killer, CD8+ memory/effector T cell, CD4+ naïve T cell,  CD4+ memory/effector T cell |
| B | CD79A, MS4A1, CD19 | 0 | 0 | 0 | Plasmacytoid dendritic | Plasmacytoid dendritic | 0 | 0 |  |
| Plasma | CD79A, CD27 | ND | ND | ND | ND | ND | ND | ND |  |
