# Supplementary material for: Evaluation of pulmonary single‐cell identity specificity in scRNA‐seq analysis
Source: Clin Transl Med. 2022 Dec 10;12(12):e1132. doi: 10.1002/ctm2.1132 (PMC9736794; doi:10.1002/ctm2.1132)
Supplement: Supplementary file 13 — Supporting Information [file CTM2-12-e1132-s009.docx]

Overlap expression cell subset of each cell subset marker gene panel of human lung tissues harvested from patients with lung adenocarcinoma (LUAD), large cell cancer (LCC), idiopathic pulmonary fibrosis (IPF), chronic obstructive pulmonary disease (COPD), and systemic sclerosis (SSC) total, normal (Norm), and para-cancer human lung tissues.

| **Cell subset** | **Gene panel** | **Total** | **Normal** | **Para-cancer** | **LCC** | **LUAD** | **IPF** | **COPD** | **SSC** |
| --- | --- | --- | --- | --- | --- | --- | --- | --- | --- |
| Natural Killer | FCER1G, TYROBP, GZMB, CHST2, HOPX | TREM2+dendritic, Signaling_AT2,  Proliferating macrophage, Platelet/Megakaryocyte  Plasmacytoid dendritic  Plasma cell,  OLR1+classic monocyte,  Nonclassical monocyte, Natural killer T cell, Myeloid dendritic type 2  Myeloid dendritic type 1, Macrophage, Ionocyte, Intermediate monocyte,  IGSF21+ dendritic, EREG+ dendritic, Classical monocyte, CD8+ naïve T cell  CD8+ memory/effector T cell, Basophil/Mast 2  Basophil/Mast 1,AT1 | TREM2+dendritic, Signaling_AT2,  Proliferating macrophage, Platelet/Megakaryocyte  Plasmacytoid dendritic  Plasma cell,  OLR1+classic monocyte,  Nonclassical monocyte, Natural killer T cell, Myeloid dendritic type 2  Myeloid dendritic type 1, Macrophage, Ionocyte, Intermediate monocyte,  IGSF21+ dendritic, EREG+ dendritic, Classical monocyte, CD8+ naïve T cell  CD8+ memory/effector T cell, Basophil/Mast 2  Basophil/Mast 1,AT1 | TREM2+dendritic, Plasmacytoid dendritic  Plasma cell, Myeloid dendritic type 2  Myeloid dendritic type 1, Macrophage, Intermediate monocyte,  IGSF21+ dendritic, EREG+ dendritic,  Classical monocyte, CD8+ naïve T cell  CD8+ memory/effector T cell, CD4+ naïve T cell  CD4+ memory/effector T cell, Capillary intermediate endothelia 1  Capillary aerocyte, Basophil/Mast 2  Basophil/Mast 1,B cell,AT2,AT1 | TREM2+dendritic, Plasmacytoid dendritic  Plasma cell, OLR1+classic monocyte,  Nonclassical monocyte, Myeloid dendritic type 2  Myeloid dendritic type 1, Macrophage, Intermediate monocyte,  IGSF21+ dendritic, EREG+ dendritic, Classical monocyte, CD8+ naïve T cell  CD8+ memory/effector T cell,  Bronchial vessel endothelia 2  Bronchial vessel endothelia 1,B cell,AT2,AT1 | TREM2+dendritic,Signaling_AT2, | 35.71 | 64.29 | 26.79 |
| Natural Killer T | CD3E, FCER1G, TYROBP | Signaling_AT2, Serous epithelia, Proliferating macrophage, Platelet/Megakaryocyte  Plasmacytoid dendritic  Plasma cell, OLR1+classic monocyte  Nonclassical monocyte, Natural killer T cell, Myeloid dendritic type 2  Myeloid dendritic type 1, Macrophage  , Intermediate monocyte  IGSF21+ dendritic, EREG+ dendritic, Classical monocyte, CD8+ naïve T cell  CD8+ memory/effector T cell, Basophil/Mast 2  Basophil/Mast 1,B cell,AT1 | Signaling_AT2, Proliferating macrophage, Platelet/Megakaryocyte  Plasmacytoid dendritic  Plasma cell, Nonclassical monocyte,  Natural killer T cell, Myeloid dendritic type 2  Myeloid dendritic type 1, Macrophage  , Intermediate monocyte  IGSF21+ dendritic, EREG+ dendritic, Classical monocyte, CD8+ naïve T cell  CD8+ memory/effector T cell, Basophil/Mast 2  Basophil/Mast 1,AT1 | TREM2+dendritic,Proliferating macrophage, Platelet/Megakaryocyte  Plasmacytoid dendritic  Plasma cell, Nonclassical monocyte,  Natural killer T cell, Myeloid dendritic type 2  Myeloid dendritic type 1, Macrophage  , Intermediate monocyte  IGSF21+ dendritic, EREG+ dendritic, Classical monocyte, CD8+ naïve T cell  CD8+ memory/effector T cell, Basophil/Mast 2  Basophil/Mast 1,AT1 | TREM2+dendritic,Proliferating macrophage, Platelet/Megakaryocyte  Plasmacytoid dendritic  Plasma cell, Nonclassical monocyte,  Natural killer T cell, Myeloid dendritic type 2  Myeloid dendritic type 1, Macrophage  , Intermediate monocyte  IGSF21+ dendritic, EREG+ dendritic, Classical monocyte, CD8+ naïve T cell  CD8+ memory/effector T cell, Basophil/Mast 2  Basophil/Mast 1,AT1 | TREM2+dendritic,Proliferating macrophage, Platelet/Megakaryocyte  Plasmacytoid dendritic  Plasma cell, Nonclassical monocyte,  Natural killer T cell, Myeloid dendritic type 2  Myeloid dendritic type 1, Macrophage  , Intermediate monocyte  IGSF21+ dendritic, EREG+ dendritic, Classical monocyte, CD8+ naïve T cell  CD8+ memory/effector T cell, Basophil/Mast 2  Basophil/Mast 1,AT1 | Signaling_AT2, Serous epithelia, Proliferating macrophage, Platelet/Megakaryocyte  Plasmacytoid dendritic  Plasma cell, OLR1+classic monocyte  Nonclassical monocyte, Natural killer T cell, Myeloid dendritic type 2  Myeloid dendritic type 1, Macrophage  , Intermediate monocyte  IGSF21+ dendritic, EREG+ dendritic, Classical monocyte, CD8+ naïve T cell  CD8+ memory/effector T cell, Basophil/Mast 2  Basophil/Mast 1,B cell,AT1 | TREM2+dendritic,Proliferating macrophage, Platelet/Megakaryocyte  Plasmacytoid dendritic  Plasma cell, Nonclassical monocyte,  Natural killer T cell, Myeloid dendritic type 2  Myeloid dendritic type 1, Macrophage  , Intermediate monocyte  IGSF21+ dendritic, EREG+ dendritic, Classical monocyte, CD8+ naïve T cell  CD8+ memory/effector T cell, Basophil/Mast 2  Basophil/Mast 1,AT1 | TREM2+dendritic,Proliferating macrophage, Platelet/Megakaryocyte  Plasmacytoid dendritic  Plasma cell, Nonclassical monocyte,  Natural killer T cell, Myeloid dendritic type 2  Myeloid dendritic type 1, Macrophage  , Intermediate monocyte  IGSF21+ dendritic, EREG+ dendritic, Classical monocyte, CD8+ naïve T cell  CD8+ memory/effector T cell, Basophil/Mast 2  Basophil/Mast 1,AT1 |
| Proliferating NK/T | MKI67, TOP2A, CD3E, FCER1G | TREM2+dendritic, Proliferating macrophage, Proliferating basal epithelia,  Platelet/Megakaryocyte,  Plasmacytoid dendritic,  Plasma cell, OLR1+classic monocyte,  Nonclassical monocyte,  Neuroendocrine epithelia,  Natural killer T cell,  Natural killer, Myeloid dendritic type 2,  Myeloid dendritic type 1,Macrophage, Ionocyte, Intermediate monocyte,  IGSF21+ dendritic, EREG+ dendritic, Classical monocyte, CD8+ naïve T cell,  CD8+ memory/effector T cell,  CD4+ naïve T cell,  CD4+ memory/effector T cell, Basophil/Mast 2  Basophil/Mast 1  ,B cell | TREM2+dendritic, Proliferating macrophage, Platelet/Megakaryocyte,  Plasmacytoid dendritic,  Plasma cell, OLR1+classic monocyte,  Nonclassical monocyte,  Neuroendocrine epithelia,  Natural killer T cell,  Natural killer,  Myofibroblast, Myeloid dendritic type 2,  Myeloid dendritic type 1,  Mucous epithelia,  Mesothelial cell,  Macrophage, Ionocyte,  Intermediate monocyte,  IGSF21+ dendritic, EREG+ dendritic,  Differentiating basal epithelia,  Club epithelia,  Classical monocyte, CD8+ naïve T cell,  CD8+ memory/effector T cell,  CD4+ naïve T cell,  CD4+ memory/effector T cell, Capillary intermediate endothelia 1, Basophil/Mast 2  Basophil/Mast 1  Basal epithelia,  B cell,AT2 | TREM2+dendritic, Plasmacytoid dendritic,  Plasma cell, OLR1+classic monocyte,  Nonclassical monocyte,  Neuroendocrine epithelia,  Natural killer T cell,  Natural killer,  Myofibroblast,  Myeloid dendritic type 2,  Myeloid dendritic type 1, Macrophage,  Lymphatic endothelia  , Intermediate monocyte,  IGSF21+ dendritic, EREG+ dendritic, Classical monocyte, Ciliated epithelia,  CD8+ naïve T cell,  CD8+ memory/effector T cell,  CD4+ naïve T cell,  CD4+ memory/effector T cell, Capillary intermediate endothelia 1, Bronchial vessel endothelia 1,  Basophil/Mast 2,  Basophil/Mast 1,  Basal epithelia,  B cell | TREM2+dendritic, Proximal basal epithelia, Platelet/Megakaryocyte,  Plasmacytoid dendritic, OLR1+classic monocyte,  Nonclassical monocyte, Natural killer T cell,  Natural killer, Myeloid dendritic type 2,  Myeloid dendritic type 1,Macrophage, Intermediate monocyte,  IGSF21+ dendritic, EREG+ dendritic, Classical monocyte, CD8+ naïve T cell,  CD8+ memory/effector T cell,  CD4+ naïve T cell,  CD4+ memory/effector T cell, Basophil/Mast 2,  Basophil/Mast 1, | TREM2+dendritic, Platelet/Megakaryocyte,  Plasmacytoid dendritic, Plasma cell, OLR1+classic monocyte,  Nonclassical monocyte, Natural killer T cell,  Natural killer, Myeloid dendritic type 2,  Myeloid dendritic type 1, Mesothelial cell,  Macrophage, Intermediate monocyte,  IGSF21+ dendritic, EREG+ dendritic, Classical monocyte, CD8+ naïve T cell,  CD8+ memory/effector T cell,  CD4+ naïve T cell,  CD4+ memory/effector T cell, Capillary intermediate endothelia 1, Basophil/Mast 2,  Basophil/Mast 1 | TREM2+dendritic, Proliferating macrophage, Platelet/Megakaryocyte,  Plasmacytoid dendritic,  Plasma cell, OLR1+classic monocyte,  Nonclassical monocyte,  Neuroendocrine epithelia,  Natural killer T cell,  Natural killer, Myeloid dendritic type 2,  Myeloid dendritic type 1,Macrophhage, Ionocyte  Intermediate monocyte,  IGSF21+ dendritic, EREG+ dendritic, Classical monocyte, CD8+ naïve T cell,  CD8+ memory/effector T cell,  CD4+ naïve T cell,  CD4+ memory/effector T cell, Basophil/Mast 2,  Basophil/Mast 1, B cell | TREM2+dendritic, Proximal ciliated epithelia, Platelet/Megakaryocyte,  Plasmacytoid dendritic,  Plasma cell, OLR1+classic monocyte,  Nonclassical monocyte, Natural killer T cell,  Natural killer, Myeloid dendritic type 2,  Myeloid dendritic type 1,  Mucous epithelia,Macrophahge, Ionocyte, Intermediate monocyte,  IGSF21+ dendritic,  Goblet epithelia,  Fibromyocyte, EREG+ dendritic, Club epithelia, Classical monocyte, CD8+ naïve T cell,  CD8+ memory/effector T cell,  CD4+ naïve T cell,  CD4+ memory/effector T cell, Capillary intermediate endothelia 1, Basophil/Mast 2  Basophil/Mast 1,B cell,AT2 | —— |
